# Transcript profiling of cytokinin action in *Arabidopsis* roots and shoots discovers largely similar but also organ-specific responses

**DOI:** 10.1186/1471-2229-12-112

**Published:** 2012-07-23

**Authors:** Wolfram G Brenner, Thomas Schmülling

**Affiliations:** 1 Institute of Biology/Applied Genetics, Dahlem Centre of Plant Sciences (DCPS), Freie Universität Berlin, Albrecht-Thaer-Weg 6, D-14195, Berlin, Germany

## Abstract

**Background:**

The plant hormone cytokinin regulates growth and development of roots and shoots in opposite ways. In shoots it is a positive growth regulator whereas it inhibits growth in roots. It may be assumed that organ-specific regulation of gene expression is involved in these differential activities, but little is known about it. To get more insight into the transcriptional events triggered by cytokinin in roots and shoots, we studied genome-wide gene expression in cytokinin-treated and cytokinin-deficient roots and shoots.

**Results:**

It was found by principal component analysis of the transcriptomic data that the immediate-early response to a cytokinin stimulus differs from the later response, and that the transcriptome of cytokinin-deficient plants is different from both the early and the late cytokinin induction response. A higher cytokinin status in the roots activated the expression of numerous genes normally expressed predominantly in the shoot, while a lower cytokinin status in the shoot reduced the expression of genes normally more active in the shoot to a more root-like level. This shift predominantly affected nuclear genes encoding plastid proteins. An organ-specific regulation was assigned to a number of genes previously known to react to a cytokinin signal, including root-specificity for the cytokinin hydroxylase gene *CYP735A2* and shoot specificity for the cell cycle regulator gene *CDKA;1*. Numerous cytokinin-regulated genes were newly discovered or confirmed, including the meristem regulator genes *SHEPHERD* and *CLAVATA1*, auxin-related genes (*IAA7*, *IAA13*, *AXR1, PIN2, PID*), several genes involved in brassinosteroid (*CYP710A1*, *CYP710A2*, *DIM/DWF*) and flavonol (*MYB12*, *CHS*, *FLS1*) synthesis, various transporter genes (e.g. *HKT1*), numerous members of the AP2/ERF transcription factor gene family, genes involved in light signalling (*PhyA*, *COP1*, *SPA1*), and more than 80 ribosomal genes. However, contrasting with the fundamental difference of the growth response of roots and shoots to the hormone, the vast majority of the cytokinin-regulated transcriptome showed similar response patterns in roots and shoots.

**Conclusions:**

The shift of the root and shoot transcriptomes towards the respective other organ depending on the cytokinin status indicated that the hormone determines part of the organ-specific transcriptome pattern independent of morphological organ identity. Numerous novel cytokinin-regulated genes were discovered which had escaped earlier discovery, most probably due to unspecific sampling. These offer novel insights into the diverse activities of cytokinin, including crosstalk with other hormones and different environmental cues, identify the AP2/ERF class of transcriptions factors as particularly cytokinin sensitive, and also suggest translational control of cytokinin-induced changes.

## Background

Cytokinin is a plant hormone regulating numerous developmental and physiological activities [1,2]. Many molecular details of cytokinin metabolism and signal transduction have been discovered during recent years. It is known that cytokinin acts locally as well as over a distance, that it is synthesized and degraded in different root and shoot tissues and that its signal is transduced through a complex two-component system. In *Arabidopsis thaliana*, three membrane-located sensor histidine kinases – AHK2, AHK3 and CRE1/AHK4 – perceive the signal and transmit it through a phosphorelay via phosphotransmitter proteins (AHP) to the nucleus where B-type response regulators (ARR), a class of transcription factors, become activated and induce the transcription of numerous target genes [3-5]. It has been shown that the B-type ARRs are involved in mediating most if not all of the transcriptional responses to cytokinin [6-8]. Genome-wide analyses of the *Arabidopsis* transcriptome have identified many genes that show a rapid up- or downregulation of their steady-state mRNA level in response to cytokinin [6,7,9-18], reviewed in [19]. Among these are numerous genes encoding transcription factors suggesting that transcriptional cascades operate downstream of cytokinin and are involved in realizing the biological output reactions. Indeed, functional studies have confirmed that beside the B-type ARRs, other factors are involved in mediating the transcriptional response to cytokinin. These include a subgroup of the ERF/AP2 transcription factor family named CYTOKININ RESPONSE FACTOR (CRF) [16], members of the GeBP transcription factor family [20] and GATA22 [21,22].

An interesting feature of cytokinin is its opposite activity in regulating growth of roots and shoots. Cytokinin is a negative regulator of root growth and branching. Plants with a reduced cytokinin status – generated either by reduction of the endogenous cytokinin content [23-26] or by disruption of cytokinin signalling [6-8,27] show enhanced root growth. In the root apical meristem, cytokinin promotes the transition from cells to differentiation [24,28] acting through the auxin-response factor IAA3/SHY2 [29]. In contrast, cytokinin retards cellular differentiation in the shoot apical meristem by interacting with the WUS/CLV pathway, as well as by a separate pathway [30-33]. Similarly, cytokinin inhibits root branching [34,35], but stimulates the growth of lateral shoots [36]. Moreover, a high cytokinin:auxin ratio favours shoot induction in undifferentiated callus tissue, while a low cytokinin:auxin ratio leads to the formation of roots [37]. In addition to these developmental changes, it has been shown that cytokinin-deficiency has different consequences on the primary metabolism of root and shoot tissue [38].

Different activities of cytokinin in the two organs could be partly realized through a different transcriptional response. Shoots and roots express specific sets of genes [39]. Here we describe the results of a genome-wide expression profiling of roots and shoots of seedlings treated with cytokinin for different periods of time. In addition, we compared the root and shoot transcriptomes of wild-type plants with those of cytokinin-deficient plants overexpressing the cytokinin oxidase/dehydrogenase gene *CKX1* under the control of the *35S* promoter [24]. We looked for similarities and differences of cytokinin-dependent gene expression in roots and shoots to identify genes with organ-specific regulation, as well as to assign a root- or shoot-specific response to already known cytokinin-regulated genes. The results of this transcript profiling approach are a starting point for further research into the differential responses of roots and shoots to cytokinin.

## Results and discussion

In order to search for short-term and long-term effects on the transcriptome, we treated 5-d-old wild-type seedlings with cytokinin for 15 min (named BA15), 2 h (BA120), and 18 h (BA1080) and harvested roots and shoots separately. To contrast the cytokinin induction, we analyzed root and shoot samples of cytokinin-deficient *35S:CKX1* expressing seedlings of the same age (named CKX1). These seedlings contain about 38% of the cytokinin levels of wild-type seedlings in the roots and about 12% in the shoots [26]. The reference samples for the whole experiment were roots and shoots of mock-treated wild-type seedlings (BA0). mRNA preparations were obtained from two biological replicates and subjected to microarray analysis as described in Methods. The hybridization strategy used for this study yielded absolute expression values instead of the fold-changes usually obtained from two-colour microarrays. To identify cytokinin-regulated genes, we considered only those transcripts which were detected on at least 25% of the microarrays (i. e. 8 microarrays for cytokinin induction and 4 microarrays for cytokinin deficiency), because inclusion of weakly expressed genes caused a large number of false positives as determined by re-examination by qRT-PCR (data not shown). In total, 8,549 and 7,557 genes were above the cutoff on the arrays used for the analysis of gene regulation by cytokinin treatment or cytokinin-deficiency, respectively. These genes were then classified as described in Methods and Additional file 1: Figure S1. Genes with more than 2.5-fold change of expression compared to the control samples are listed in Additional file 2: Table S1 (cytokinin induction, 1,450 genes) and Additional file 3: Table S2 (cytokinin deficiency, 1,473 genes), respectively. Selected examples of these genes are listed in Table 1 (cytokinin induction) and Table 2 (cytokinin deficiency) and will be commented on further below.

**Table 1:** Organ-specific changes of transcript abundance in response to cytokinin treatment

**Category**	**CATMA ID**	**AbB**	**Ratio**						**FVR p-value cytokinin effect**	**AGI**	**Description**
			**Root**			**Shoot**					
			**BA15 vs. BA0**	**BA120 vs. BA0**	**BA1080 vs. BA0**	**BA15 vs. BA0**	**BA120 vs. BA0**	**BA1080 vs. BA0**			
root-specific	CATMA1a44090	9	**5.71**	**75.18**	**156.88**	0.74	0.47	0.52	**1.81E-02**	AT1G53060	legume lectin family protein
	CATMA1a56420	8	**4.19**	**60.47**	**29.56**	0.90	0.62	1.50	**4.72E-03**	AT1G67110	cytochrome P450, putative
	CATMA2a32630	11	1.68	*0.26*	**66.01**	1.61	0.55	0.83	**3.02E-07**	AT2G34490	cytochrome P450 family protein
	CATMA4a38980	13	**6.85**	**21.89**	**16.03**	1.50	1.03	0.99	**4.49E-03**	AT4G37410	cytochrome P450, putative
	CATMA3a15410	8	0.89	**19.41**	**3.14**	1.55	1.47	0.71	**2.38E-03**	AT3G15990	sulfate transporter, putative
	CATMA1a29550	13	0.97	**6.93**	**9.80**	0.82	0.89	1.93	**1.25E-04**	AT1G31320	LOB domain protein 4 (LBD4)
	CATMA5a61330	18	**2.56**	**8.17**	**5.68**	1.21	0.84	1.02	**5.89E-04**	AT5G65990	amino acid transporter family protein
	CATMA4a30310	28	0.99	0.86	**5.08**	0.65	0.54	0.81	**1.31E-06**	AT4G28660	photosystem II reaction centre W (PsbW) family protein
	CATMA5a50360	21	0.49	0.64	**5.65**	1.00	0.56	1.16	**6.90E-06**	AT5G54510	IAA-amido synthase
	CATMA3a26362	30	0.93	0.51	**4.73**	0.98	0.69	1.53	**1.35E-05**	AT3G26570	phosphate transporter family protein
	CATMA2a00475	24	1.15	1.12	**3.41**	0.62	0.61	0.55	**1.67E-02**	AT2G01420	auxin transport protein, putative
	CATMA4a22880	19	0.86	0.45	**2.71**	0.66	0.46	0.54	**2.32E-04**	AT4G21280	PsbQ subunit of photosystem II.
	CATMA4a33000	9	1.04	0.64	*0.07*	1.12	1.79	1.43	**7.04E-04**	AT4G31320	auxin-responsive protein, putative (SAUR_C)
	CATMA4a20925	12	0.95	*0.35*	*0.04*	1.39	0.74	0.71	**3.39E-04**	AT4G19690	iron-responsive transporter (IRT1)
	CATMA4a11410	6	0.82	*0.24*	*0.08*	0.93	1.14	0.97	**1.09E-02**	AT4G11280	1-aminocyclopropane-1-carboxylate (ACC) synthase
shoot-specific	CATMA5a49210	6	2.28	1.71	0.79	**5.42**	**7.91**	**6.22**	**2.53E-05**	AT5G53290	ERF subfamily B-5 of ERF/AP2 transcription factor
	CATMA2a30720	12	0.82	2.11	0.71	**5.49**	**6.48**	**7.33**	**7.58E-03**	AT2G32430	galactosyltransferase family protein
	CATMA5a51700	7	1.48	1.64	0.43	**3.18**	**9.71**	**5.61**	**5.80E-03**	AT5G55920	nucleolar protein, putative
	CATMA3a41735	22	1.00	1.37	0.64	**8.68**	**7.88**	1.83	1.21E-01	AT3G48750	A-type cyclin-dependent kinase
	CATMA4a19065	19	2.17	1.45	0.48	**3.05**	**6.26**	**4.66**	**1.09E-02**	AT4G18040	eukaryotic translation initiation factor 4E 1 (EIF4E1)
	CATMA3a10615	6	0.72	1.35	0.61	**2.91**	**5.89**	**3.86**	**2.37E-03**	AT3G11670	digalactosyldiacylglycerol synthase 1 (DGD1)
	CATMA1a28980	22	0.57	0.50	1.51	**3.39**	1.78	2.26	**1.19E-02**	AT1G30840	purine permease-related
	CATMA2a32773	9	1.19	1.14	1.69	0.83	*0.34*	*0.34*	**2.80E-02**	AT2G34650	protein kinase PINOID (PID)
	CATMA5a53690	31	0.85	0.63	1.80	0.53	0.45	*0.39*	**8.23E-03**	AT5G57930	ACCUMULATION OF PHOTOSYSTEM ONE 2 (APO2)
	CATMA1a65110	29	1.04	0.79	0.54	*0.34*	*0.32*	*0.09*	**3.96E-07**	AT1G75820	CLAVATA1 receptor kinase (CLV1)
	CATMA5a43215	30	0.50	0.80	0.87	0.44	*0.18*	*0.08*	**3.27E-03**	AT5G47230	ERF subfamily B-3 of ERF/AP2 transcription factor (ATERF-5)
	CATMA4a28860	31	0.53	0.50	0.85	*0.26*	*0.17*	*0.07*	**8.86E-05**	AT4G27280	calcium-binding EF hand family protein
differential	CATMA4a13955	22	1.87	1.07	*0.22*	**10.41**	**4.16**	**2.68**	**2.25E-04**	AT4G13770	cytochrome P450 family protein
	CATMA5a03540	10	0.71	**2.78**	**5.07**	0.44	*0.18*	0.50	**1.01E-02**	AT5G04330	cytochrome P450, putative
similar	CATMA5a07985	17	**2.96**	2.22	1.85	**14.50**	**30.04**	**4.43**	**2.03E-02**	AT5G08640	flavonol synthase 1 (FLS1)
	CATMA3a18390	5	**2.90**	**12.10**	**4.22**	**9.20**	**4.14**	**2.85**	**1.05E-03**	AT3G18773	zinc finger (C3HC4-type RING finger) family protein
	CATMA2b16180	13	**2.72**	**12.21**	**8.03**	**2.92**	**2.72**	**2.64**	**7.70E-03**	AT2G17500	auxin efflucarrier family protein
	CATMA4a41340	17	**2.58**	2.26	**3.97**	**5.32**	**4.92**	**10.72**	**1.08E-04**	AT4G39950	cytochrome P450 79B2, putative (CYP79B2)
	CATMA1a18090	15	**3.90**	**12.44**	0.83	1.90	**6.88**	1.54	**4.74E-06**	AT1G19050	response regulator 7 (ARR7)
	CATMA2a16180	11	1.01	**7.02**	**5.94**	**3.08**	1.61	1.85	**1.01E-02**	AT2G17500	auxin efflucarrier family protein
	CATMA4a25920	22	**3.39**	2.18	0.72	**3.05**	**7.24**	2.36	**1.25E-04**	AT4G24190	shepherd protein (SHD)
	CATMA4a20920	12	1.07	1.57	**3.05**	1.03	**4.11**	**7.38**	**1.47E-02**	AT4G19680	iron-responsive transporter (IRT2)
	CATMA5a46780	4	0.81	**4.71**	**2.55**	2.12	**2.52**	**4.32**	**1.40E-02**	AT5G50820	no apical meristem (NAM) family protein
	CATMA5a19900	9	**4.76**	2.40	1.61	**2.80**	**3.08**	2.25	**2.14E-02**	AT5G22440	60S ribosomal protein L10A (RPL10aC)
	CATMA2a29850	28	**2.70**	2.11	**3.60**	**2.93**	**2.57**	**2.62**	**8.25E-03**	AT2G31610	40S ribosomal protein S3 (RPS3A)
	CATMA1a69100	20	**3.03**	**2.52**	2.38	2.11	**4.75**	1.72	**1.99E-02**	AT1G79920	heat shock protein 70, putative (HSP70)
	CATMA5a43680	27	**2.66**	1.68	**3.14**	**3.01**	**3.43**	2.48	**1.43E-02**	AT5G47700	60S acidic ribosomal protein P1 (RPP1C)
	CATMA4a31370	15	0.43	**4.28**	**3.45**	1.19	1.89	**4.42**	**4.75E-05**	AT4G29740	FAD-binding domain-containing protein
	CATMA1a48985	6	0.84	**3.57**	**4.63**	2.27	1.03	**3.28**	**2.86E-03**	AT1G59940	response regulator (ARR)
	CATMA3a45520	17	**2.65**	1.44	**2.74**	1.34	2.27	**2.94**	**1.95E-02**	AT3G52580	40S ribosomal protein S14 (RPS14C)
	CATMA1a09860	13	2.25	1.17	**3.18**	**3.46**	1.96	0.54	**7.61E-03**	AT1G10970	metal transporter, putative (ZIP4)
	CATMA3a48290	12	**2.71**	1.42	0.86	**4.96**	2.26	0.96	**1.77E-02**	AT3G55280	60S ribosomal protein L23A (RPL23aB)
	CATMA2a26520	26	0.78	*0.27*	1.61	1.14	*0.40*	0.74	**8.72E-05**	AT2G28120	nodulin family protein
	CATMA4a08040	24	0.80	0.58	*0.31*	1.74	1.08	*0.23*	**1.41E-05**	AT4G08290	nodulin MtN21 family protein
	CATMA4a01650	8	1.07	1.03	*0.17*	1.13	0.81	*0.39*	**9.55E-03**	AT4G01450	nodulin MtN21 family protein
	CATMA2a31475	31	*0.29*	0.82	1.51	*0.39*	0.46	0.59	**3.36E-03**	AT2G33310	indoleacetic acid-induced protein 13 (IAA13)
	CATMA1a01400	10	0.62	0.84	*0.26*	1.12	*0.36*	*0.27*	**2.11E-02**	AT1G02400	gibberellin 2-oxidase
	CATMA4a08050	23	0.52	*0.30*	*0.27*	1.10	0.83	*0.16*	**9.93E-06**	AT4G08300	nodulin MtN21 family protein
	CATMA2a25030	24	0.56	*0.39*	0.56	0.64	0.64	*0.34*	**4.80E-03**	AT2G26710	cytochrome p450 family
	CATMA1a18780	31	0.77	*0.27*	0.80	0.73	*0.31*	*0.22*	**6.34E-05**	AT1G19770	purine permease-related
	CATMA3a22995	31	0.63	*0.37*	0.57	*0.35*	0.48	0.69	**1.67E-02**	AT3G23050	indoleacetic acid-induced protein 7 (IAA7)
	CATMA5a59720	22	0.57	*0.27*	0.78	0.51	*0.29*	*0.19*	**1.55E-04**	AT5G64260	phosphate-responsive protein, putative
	CATMA1a66768	9	1.01	0.46	*0.24*	0.62	*0.20*	*0.06*	**8.11E-05**	AT1G77640	DREB subfamily A-5 of ERF/AP2 transcription factor
	CATMA1a33270	21	0.78	*0.11*	*0.16*	0.55	*0.27*	0.60	**6.69E-05**	AT1G35140	phosphate-responsive protein
	CATMA3a25040	24	0.82	0.52	*0.13*	0.45	*0.38*	*0.06*	**7.64E-07**	AT3G25190	nodulin, putative
	CATMA3a14910	27	0.51	*0.30*	0.60	0.49	*0.22*	*0.14*	**2.37E-04**	AT3G15500	no apical meristem (NAM) family protein (NAC3)
similar	CATMA5a57200	28	0.65	*0.33*	0.60	*0.32*	*0.12*	*0.09*	**9.30E-05**	AT5G61600	ERF subfamily B-3 of ERF/AP2 transcription factor
	CATMA1a26550	17	0.71	*0.19*	*0.09*	0.69	*0.22*	*0.12*	**1.61E-05**	AT1G28370	ERF subfamily B-1 of ERF/AP2 transcription factor
	CATMA3a14565	31	0.41	*0.17*	0.70	0.40	*0.14*	*0.17*	**3.42E-04**	AT3G15210	ERF subfamily B-1 of ERF/AP2 transcription factor (ATERF-4)
	CATMA5a47120	23	0.65	*0.30*	*0.33*	0.43	*0.11*	*0.08*	**2.60E-04**	AT5G51190	ERF subfamily B-3 of ERF/AP2 transcription factor
	CATMA5a43205	26	0.60	0.43	*0.11*	0.47	*0.18*	*0.07*	**2.68E-06**	AT5G47220	ERF subfamily B-3 of ERF/AP2 transcription factor
	CATMA2a43300	20	0.42	0.64	*0.21*	*0.36*	*0.15*	*0.05*	**5.73E-04**	AT2G44840	ERF subfamily B-3 of ERF/AP2 transcription factor
	CATMA3a54340	27	0.41	*0.16*	*0.26*	*0.23*	*0.24*	*0.14*	**2.27E-03**	AT3G61190	BON1-associated protein 1 (BAP1)
	CATMA5a06800	26	0.85	0.44	*0.40*	0.90	0.68	*0.34*	**1.93E-06**	AT5G07580	ERF subfamily B-3 of ERF/AP2 transcription factor
uncategorized	CATMA2a39655	8	0.93	2.15	*0.27*	**2.78**	**13.61**	**2.65**	**2.87E-02**	AT2G41310	response regulator 3 (ARR3)
	CATMA1a08395	15	0.58	0.52	1.31	*0.32*	*0.37*	*0.39*	**1.12E-02**	AT1G09530	phytochrome interacting factor 3 (PIF3)
	CATMA2a37175	9	**2.69**	**4.10**	0.81	2.50	1.54	0.56	**1.77E-02**	AT2G38940	phosphate transporter (PT2)
	CATMA1a68670	23	1.63	1.43	1.36	2.45	**3.23**	**3.06**	**2.54E-02**	AT1G79560	FtsH protease
	CATMA5a43410	15	0.48	*0.18*	*0.00*	**3.30**	0.70	*0.10*	**1.31E-06**	AT5G47450	major intrinsic family protein
	CATMA2a31585	30	1.06	0.59	*3.55*	**0.60**	0.50	*1.29*	**3.99E-05**	AT2G33430	plastid developmental protein DAG
	CATMA2a34435	28	0.96	*1.00*	*2.51*	**0.86**	1.27	*1.22*	**9.89E-05**	AT2G36250	chloroplast division protein FtsZ (FtsZ2-1)
	CATMA5a43410	15	0.48	*0.18*	*0.00*	**3.30**	0.70	*0.10*	**1.31E-06**	AT5G47450	major intrinsic family protein

The table contains an arbitrary selection of genes showing an organ-specific change of transcript abundance in response to cytokinin treatment. A number of these genes are discussed in the text. The categorization of genes was performed as described in Methods and in Additional file 1: Figure S1. Expression ratios in roots and shoots at different time points in relation to the control are given and coded by different font variants: bold, upregulation; italics, downregulation; regular, no regulation. CATMA ID, unique identifier for the gene specific tag (probe) on the microarray (data can be queried under http://www.catma.org); AbB, number of spots above background (a rough measure for expression strength); AGI, unique gene identifier assigned by TAIR; Description, edited TAIR database annotation. FDR corrected p-values ≤ 0.03, indicating whether a gene was significantly regulated by cytokinin, are marked with bold numbers.

**Table 2:** Organ-specificity of genes differentially expressed in cytokinin-deficient plants

**Category**	**CATMA ID**	**AbB**	**Ratio CKX1 vs. Col-0**		**FDR p-value genotype effect**	**AGI**	**Description**
			**Root**	**Shoot**			
root-specific	CATMA5a61330	11	**18.32**	1.26	**7.99E-04**	AT5G65990	amino acid transporter family protein
	CATMA3a43475	5	**7.63**	1.57	**9.62E-06**	AT3G50410	Dof-type zinc finger domain-containing protein
	CATMA1a62410	4	**5.66**	0.63	**4.99E-02**	AT1G73165	Clavata3 / ESR-Related-1 (CLE1)
	CATMA5a37900	7	**4.72**	1.07	**4.77E-03**	AT5G42200	zinc finger (C3HC4-type RING finger) family protein
	CATMA3a40595	4	**2.86**	0.97	**5.65E-03**	AT3G47600	myb family transcription factor (MYB94)
	CATMA1a66990	9	**2.60**	0.89	**5.31E-03**	AT1G77840	eukaryotic translation initiation factor 5, putative (eIF-5)
	CATMA4a36496	11	*0.27*	1.10	**1.04E-02**	AT4G34680	GATA transcription factor 3, putative (GATA-3)
	CATMA2a45310	4	*0.02*	1.03	**1.77E-02**	AT2G46870	DNA-binding protein, putative
shoot-specific	CATMA4a00090	9	1.00	**76.53**	**1.19E-03**	AT4G00080	invertase/pectin methylesterase inhibitor family protein
	CATMA5a49170	9	1.33	**15.44**	**4.78E-03**	AT5G53250	arabinogalactan-protein, putative (AGP22)
	CATMA4a26210	10	1.46	**12.59**	**1.52E-06**	AT4G24480	serine/threonine protein kinase, putative
	CATMA4a02865	9	1.01	**8.82**	**2.70E-03**	AT4G02560	homeobox protein LUMINIDEPENDENS (LD)
	CATMA3a15410	4	0.55	**8.43**	**1.83E-02**	AT3G15990	sulfate transporter, putative
	CATMA2a44715	11	1.63	**4.29**	**4.50E-04**	AT2G46340	phytochrome A supressor spa1 (SPA1)
	CATMA2a31150	13	0.68	**3.08**	**1.72E-02**	AT2G32950	COP1 regulatory protein
	CATMA2a00475	14	1.07	*0.38*	**2.37E-02**	AT2G01420	auxin transport protein, putative
	CATMA1a08416	13	0.81	*0.33*	**2.02E-02**	AT1G09570	phytochrome A (PHYA)
	CATMA3a19415	16	1.24	*0.33*	**2.33E-02**	AT3G19820	cell elongation protein DWARF1 / DIMINUTO (DWF1/DIM)
	CATMA5a06800	13	0.59	*0.20*	**2.63E-05**	AT5G07580	ERF subfamily B-3 of ERF/AP2 transcription factor
	CATMA4a18523	10	0.72	*0.20*	**2.23E-03**	AT4G17490	ERF subfamily B-3 of ERF/AP2 transcription factor (ATERF-6)
	CATMA2a32570	16	0.69	*0.18*	**1.82E-03**	AT2G34420	chlorophyll A-B binding protein (LHB1B2)
	CATMA4a36460	15	0.99	*0.17*	**3.19E-03**	AT4G34620	ribosomal protein S16 family protein
	CATMA2a24825	13	1.86	*0.16*	**1.26E-02**	AT2G26500	cytochrome b6f complex subunit (petM), putative
	CATMA5a60355	12	0.41	*0.15*	**2.94E-06**	AT5G64920	COP1-interacting protein (CIP8)
	CATMA4a22880	9	1.10	*0.15*	**4.50E-03**	AT4G21280	PsbQ subunit of photosystem II
	CATMA5a59535	4	0.59	*0.12*	**1.99E-04**	AT5G64040	photosystem I reaction center subunit PSI-N
	CATMA1a68150	7	0.70	*0.11*	**8.69E-03**	AT1G79040	10 kDa PsbR subunit of photosystem II (PSII)
	CATMA5a43205	16	0.63	*0.11*	**1.37E-05**	AT5G47220	ERF subfamily B-3 of ERF/AP2 transcription factor (ATERF-2)
	CATMA1a68150	7	0.70	*0.11*	**8.69E-03**	AT1G79040	10 kDa PsbR subunit of photosystem II
	CATMA1a46750	13	1.38	*0.06*	**3.91E-03**	AT1G55670	photosystem I reaction center subunit V
	CATMA5a43215	15	0.78	*0.05*	**7.70E-04**	AT5G47230	ERF subfamily B-3 of ERF/AP2 transcription factor (ATERF-5)
	CATMA2a43300	11	0.52	*0.04*	**9.62E-06**	AT2G44840	ERF subfamily B-3 of ERF/AP2 transcription factor
	CATMA5a57200	11	0.64	*0.03*	**3.30E-03**	AT5G61600	ERF subfamily B-3 of ERF/AP2 transcription factor
	CATMA5a47120	7	0.42	*0.03*	**1.20E-03**	AT5G51190	ERF subfamily B-3 of ERF/AP2 transcription factor
similar	CATMA5a07985	10	**16.98**	**57.30**	**3.42E-03**	AT5G08640	flavonol synthase 1 (FLS1)
	CATMA4a10345	8	**24.01**	**16.17**	**1.99E-03**	AT4G10310	sodium transporter (HKT1)
	CATMA5a52815	11	**3.38**	**33.99**	**5.78E-05**	AT5G57090	auxin transport protein (EIR1) (PIN2)
	CATMA2a45915	8	**4.11**	**26.54**	**5.82E-04**	AT2G47460	subgroup 7 of R2R3-MYB family (MYB12)
	CATMA2a32640	11	**3.20**	**25.43**	**2.07E-04**	AT2G34500	cytochrome P450 family protein
	CATMA1a04035	5	**3.86**	**5.95**	**1.72E-02**	AT1G05180	auxin-resistance protein (AXR1)
	CATMA5a12150	15	**3.65**	**4.48**	**2.57E-04**	AT5G13930	chalcone synthase
	CATMA3a11725	8	**4.38**	**3.17**	**1.42E-02**	AT3G12750	zinc transporter (ZIP1)
	CATMA3a54340	14	*0.34*	*0.29*	**2.75E-02**	AT3G61190	BON1-associated protein 1 (BAP1)
	CATMA1a26550	4	*0.34*	*0.26*	**9.57E-06**	AT1G28370	ERF subfamily B-1 of ERF/AP2 transcription factor
	CATMA1a09322	10	*0.30*	*0.29*	**1.19E-03**	AT1G10470	response regulator 4 (ARR4)
	CATMA3a16175	16	*0.17*	*0.38*	**3.92E-03**	AT3G16770	ERF subfamily B-2 of ERF/AP2 transcription factor (RAP2.3)
	CATMA5a04585	11	*0.21*	*0.31*	**7.30E-03**	AT5G05410	DREB subfamily A-2 of ERF/AP2 transcription factor (DREB2A)
	CATMA3a14565	15	*0.26*	*0.20*	**4.00E-03**	AT3G15210	ERF subfamily B-1 of ERF/AP2 transcription factor (ATERF-4)
	CATMA5a58475	5	*0.11*	*0.29*	**6.22E-04**	AT5G62920	response regulator (ARR6)
	CATMA1a66910	8	*0.28*	*0.09*	**7.72E-04**	AT1G77760	nitrate reductase 1 (NR1)
	CATMA1a57435	6	*0.12*	*0.18*	**1.18E-03**	AT1G68050	F-box family protein (FKF1) / adagio 3 (ADO3)
	CATMA1b35155	7	*0.19*	*0.05*	**8.13E-04**	AT1G37130	nitrate reductase 2 (NR2)

The table contains an arbitrary selection of genes showing altered transcript abundance under cytokinin deficiency. A number of these genes are discussed in the text. The categorization was performed as described in Methods and in Additional file 1: Figure S1. Expression ratios in roots and shoots of cytokinin-deficient seedlings relative to the wild-type control are given and coded by font variants: bold, upregulation; italics, downregulation; regular, no regulation. CATMA ID, unique identifier for the gene specific tag (probe) on the microarray (data can be queried under http://www.catma.org); AbB, number of spots above background (a rough measure for expression strength); AGI, unique gene identifier assigned by TAIR; Description, edited TAIR database annotation. FDR corrected p-values ≤ 0.03, indicating whether a gene was significantly regulated by cytokinin, are marked with bold numbers.

### Global expression analysis by principal component analysis

In order to get an insight into the global structure of the complex dataset of this microarray investigation, we performed a principal component analysis (PCA) on a reduced dataset (see Methods) using the steady-state mRNA levels determined by the application of a common reference hybridization strategy (see Methods). This type of analysis applied to the samples of a microarray experiment typically results in clusters of samples with a similar expression pattern, thereby revealing the main factors that lead to changes in gene expression. Additionally, we performed a support tree (ST) clustering, which served as a confirmation of the PCA.

### Cytokinin deficiency has a large and specific influence on the transcriptome

**Figure 1:**
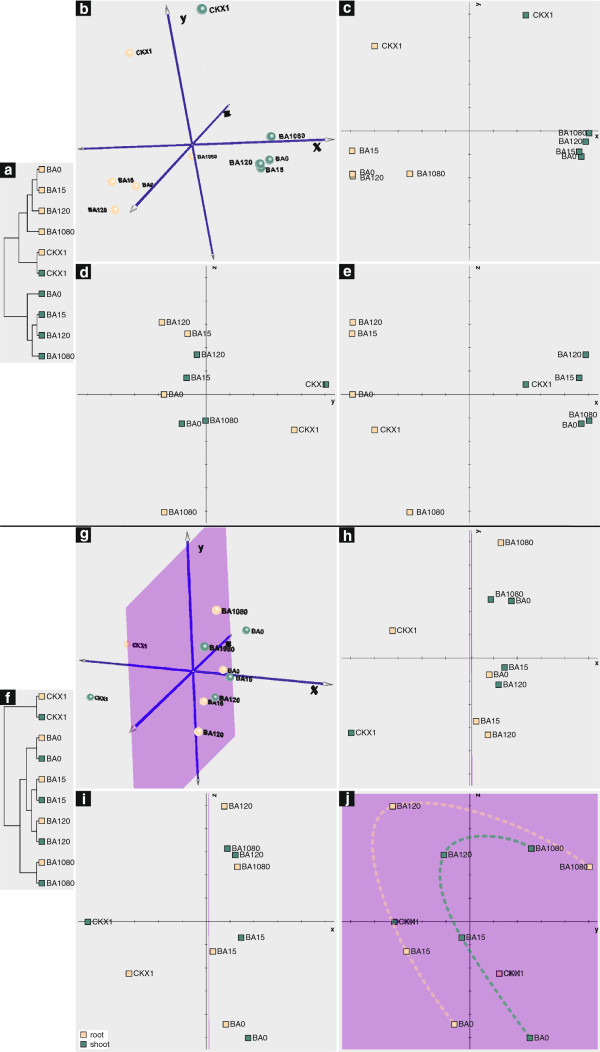
**Clustering and principal component analysis of the samples used for transcript profiling.** The analysis was carried out with 911 cytokinin-regulated genes that were selected as described in Methods. (**a**-**e**) Analysis using the native dataset. (**f**-**j**) Analysis using the dataset after normalization for the organ effect. (**a**, **f**) Support tree clustering for the samples. (**b**-**e**, **g**-**h**) Results of the principal component analyses. (**b**, **g**) Three-dimensional representation of the results of the principal component analyses. (**c**-**e**, **h**-**j**) Two-dimensional plots showing each possible combination of the three dimensions. (**c**, **h**) Plots centred on the x-axis; (**d**, **i**) plots centred on the y-axis; (**e**, **j**) plots centred on the z-axis of (**b**) and (**g**), respectively. Each point represents an experimental condition, averaging two microarray replicates of two biological replicates. BA0, control; BA15, 15 min of cytokinin treatment; BA120, 2 h of cytokinin treatment; BA1080, 18 h of cytokinin treatment; CKX1, cytokinin oxidase/dehydrogenase overexpressors. Green, shoot samples; ochre, root samples. The purple markings represent the plane on which the wild-type samples are arranged. The dashed lines in (**j**) show the factors of global gene expression changes in roots and shoots, respectively. The eigenvalues, which indicate how much of the total variation of the dataset is covered by the respective axis are for b-e: x, 0.490; y, 0.184; z, 0.112; and for g-h: x, 0.269; y, 0.239; z, 0.179. A PCA with a more restricted dataset containing 637 genes (i.e. all genes detected on at least 50% of the arrays) yielded a similar result (data not shown).

The results of the PCA show a separation of the dataset into distinct clusters (Figure 1b-e). As expected, the largest distance was found between root and shoot samples confirming the functionality of the PCA (Figure 1b, c, e). The samples of the cytokinin-deficient plants were separated from the wild-type samples on a different axis than the organ effect (Figure 1b – d). Additionally, they form their own cluster in ST clustering (Figure 1a). The large distance of the cytokinin-deficient samples from the wild-type clusters means that cytokinin deficiency causes a global transcript change to an extent comparable to the difference between roots and shoots.

Taken together, PCA analysis groups the samples into four clusters — wild-type roots, wild-type shoots, cytokinin-deficient roots, and cytokinin-deficient shoots — each of which occupies one quadrant of the clustering space (Figure 1b-e).

### The cytokinin status influences the organ-specific transcriptome pattern

Closer examination of the distribution of the samples within the clustering space revealed that a long-term cytokinin treatment of roots and constitutively lowered cytokinin content in shoots causes distinct changes of the gene expression pattern shifting the location of the root and shoot samples in the PCA towards the respective other organ.

**Figure 2:**
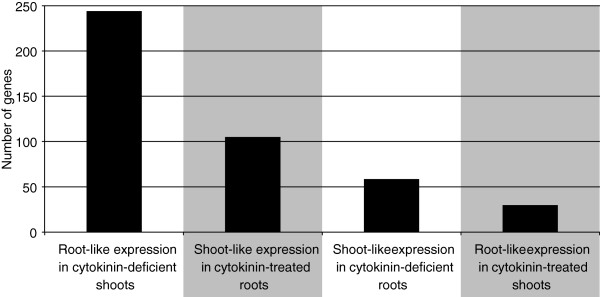
**Numbers of genes indicative of a developmental shift.** The diagram shows the numbers of genes regulated in fashion of the respective other organ in response to long-term cytokinin treatment or chronic cytokinin deficiency. Genes were selected according to the following criteria: They were differentially expressed in untreated roots and shoots, their expression level was altered ≥ 2.5-fold after 18 h of cytokinin treatment, and their p-values of the organ, cytokinin and/or interaction effects were ≤ 0.03.

Firstly, the long-term cytokinin-treated root sample is located more closely to the shoot coordinates than any other root sample (Figure 1b, c, e). This change indicates that after 18 h of cytokinin treatment, a shift in gene expression towards a shoot-like pattern has started without gross visible developmental changes having occurred in the tissue. Next, we looked for affected genes and identified these based on their differential expression between untreated roots and shoots (≥ 2.5-fold), their altered expression level after 18 h of cytokinin treatment (≥ 2.5-fold), and by their p-values of the organ, cytokinin and/or interaction effects (≤ 0.03). The transcripts of 106 genes are regulated in this fashion (Figure 2, Additional file 4: Table S3). Among the 59 genes that are upregulated towards a more shoot-like transcript level in long-term cytokinin-treated roots, we identified genes encoding chloroplast-localized proteins as the major overrepresented gene ontology class (70% of the regulated genes) (Additional file 5: Figure S2a, Additional file 4: Table S3a). These include genes encoding proteins necessary for plastid proliferation, such as the plastid developmental protein DAG (AT2G33430) and the chloroplast division protein FtsZ (AT2G36250). Conversely, 47 predominantly root-expressed genes, such as AT5G47450 encoding the tonoplast intrinsic protein TIP2;3 transporting ammonium across the tonoplast (Table 1, Additional file 4: Table S3b, Additional file 6: Figure S3a, b, Additional file 7: Figure S4a, b) [40], are repressed by long-term cytokinin treatment in roots. However, no overrepresented functional GO category could be found among this group of genes. In comparison, only 30 genes acquired a more root-like expression upon cytokinin treatment of shoots (Figure 2) and no shift of the respective sample towards root-like expression was observed in the PCA (Figure 1b-e). No GO category could be found to be overrepresented among these genes.

Secondly, a similar, albeit opposite, developmental shift effect was observed in the shoot sample of cytokinin-deficient plants, which is located more closely to the root coordinates than any other shoot sample (Figure 1a - c, e). This global shift of the transcript profile to a more root-like pattern suggests that, beside the visible reduction of shoot growth, a low cytokinin status reduces the “shootyness” of the global transcription pattern. We identified genes displaying a corresponding expression pattern as described above. 246 genes show a more root-like transcript level in shoots of cytokinin-deficient seedlings (Figure 2, Additional file 8: Table S4). Also in this case, the set of 169 genes that was downregulated to a more root-like transcript level contains a high fraction (52%) of chloroplast-related genes (Additional file 5: Figure S2b, Additional file 8: Table S4b). The gene encoding the PsbR subunit of photosystem II (AT1G79040), for example, is downregulated to about one tenth of its expression level in shoots of cytokinin-deficient plants. Conversely, 77 otherwise predominantly shoot-expressed genes are stronger expressed in cytokinin-deficient roots (Figure 2, Additional file 8: Table S4a), such as *PIN2*, encoding a root-specific auxin efflux carrier discussed further below, but no functional GO category was found to be overrepresented among them.

Interestingly, 63 of all regulated genes showing an enhanced transcript level in cytokinin-treated roots were also repressed in cytokinin-deficient shoots and vice versa indicating that these are particularly cytokinin-sensitive genes (Table 3). 34 of the 44 genes enhanced by cytokinin in roots and repressed in cytokinin-deficient shoots encode plastid proteins with functions e.g. in the light reaction or the calvin cycle as well as several plastidal ribosomal proteins (Table 3). Examples of the 19 genes repressed by cytokinin in roots and enhanced in cytokinin-deficient shoots encode the predominantly root-expressed sodium transporter HKT1 discussed further below, and the signalling protein CBL-interacting protein kinase 23 (CIPK23, AT1G30270).

**Table 3:** Genes indicative of a developmental shift due to an altered cytokinin status

**CATMA ID**	**AbB**	**Ratio**			**FDR p-value**			**AGI**	**Subcellular localization**	**Description**
		**BA1080 vs. BA0 Root**	**CKX1 vs. BA0 Shoot**	**Root vs. Shoot @ BA0**	**Cytokinin effect**	**Organ effect**	**Interaction effect**			
CATMA2a26583	40	**2.71**	*0.09*	*0.04*	**5.78E-07**	**3.27E-11**	**1.12E-06**	AT2G28190	a,P	superoxide dismutase (Cu-Zn) (SODCP)
CATMA5a16150	35	**2.80**	*0.09*	*0.03*	**1.83E-06**	**5.98E-18**	**2.95E-05**	AT5G17870	P	plastid-specific ribosomal protein-related
CATMA3a44603	29	**2.71**	*0.12*	*0.00*	**4.68E-03**	**2.58E-21**	**5.67E-05**	AT3G51600	w	nonspecific lipid transfer protein 5 (LTP5)
CATMA1a65310	37	**4.44**	*0.14*	*0.01*	**3.01E-11**	**1.64E-29**	**2.54E-10**	AT1G76080	P	thioredoxin family protein
CATMA2a35500	34	**3.08**	*0.13*	*0.07*	**2.34E-06**	**5.32E-13**	**2.70E-04**	AT2G37220	P	29 kDa ribonucleoprotein
CATMA1a13920	37	**2.76**	*0.13*	*0.20*	**8.52E-05**	**7.32E-05**	**4.18E-04**	AT1G14890	—	invertase/pectin methylesterase inhibitor family protein
CATMA1a19333	37	**3.21**	*0.14*	*0.01*	**3.04E-06**	**6.27E-22**	**9.90E-06**	AT1G20340	P	plastocyanin
CATMA4a22880	23	**2.73**	*0.14*	*0.01*	**3.41E-06**	**5.25E-25**	**7.49E-06**	AT4G21280	P	PsbQ subunit of photosystem II
CATMA2a04295	38	**2.74**	*0.15*	*0.03*	**8.82E-04**	**3.72E-19**	**6.27E-06**	AT2G05520	e	glycine-rich protein (GRP)
CATMA5a07300	33	**6.42**	*0.35*	*0.01*	**5.78E-05**	**2.59E-21**	**1.92E-06**	AT5G08050	P	expressed protein
CATMA5a12555	27	**3.74**	*0.18*	*0.02*	**8.15E-06**	**1.76E-21**	**2.42E-06**	AT5G14320	P	30S ribosomal protein S13, chloroplast (CS13)
CATMA4a30310	36	**5.11**	*0.25*	*0.01*	**3.41E-06**	**6.23E-24**	**4.24E-06**	AT4G28660	P	photosystem II reaction centre W (PsbW) family protein
CATMA3b54606	36	**2.96**	*0.17*	*0.01*	**2.16E-02**	**1.77E-21**	**5.67E-05**	AT3G61470	P	chlorophyll A-B binding protein (LHCA2)
CATMA3a14550	40	**3.01**	*0.19*	*0.04*	**7.16E-06**	**4.96E-20**	**3.23E-04**	AT3G15190	P	chloroplast 30S ribosomal protein S20
CATMA2a41430	36	**3.74**	*0.22*	*0.04*	**3.50E-05**	**4.69E-18**	**2.18E-04**	AT2G43030	P	ribosomal protein L3 family protein
CATMA4a26750	18	**2.92**	*0.19*	*0.03*	**1.59E-05**	**2.30E-20**	**8.52E-05**	AT4G25050	P	acyl carrier protein predominantly expressed in leaves
CATMA2a35140	30	**3.69**	*0.23*	*0.03*	**2.86E-05**	**4.06E-18**	**9.10E-04**	AT2G36870	a,e,w	xyloglucan:xyloglucosyl transferase
CATMA1a63880	31	**3.32**	*0.22*	*0.04*	**7.39E-04**	**2.67E-18**	**9.61E-05**	AT1G74470	P	geranylgeranyl reductase
CATMA4a18000	32	**4.95**	*0.35*	*0.01*	**2.52E-06**	**1.37E-22**	**8.09E-06**	AT4G16980	e	arabinogalactan-protein family
CATMA5a51450	30	**2.74**	*0.20*	*0.05*	**1.20E-03**	**8.97E-17**	**7.88E-05**	AT5G55700	P	similar to beta-amylase (CT-BMY)
CATMA4a40410	32	**2.58**	*0.19*	*0.01*	**1.30E-05**	**8.94E-23**	**1.90E-04**	AT4G38970	a,P	fructose-bisphosphate aldolase
CATMA2a37967	40	**2.68**	*0.20*	*0.02*	**1.15E-05**	**2.09E-22**	**3.60E-05**	AT2G39730	a,n,P,w	RuBisCO activase
CATMA1a36300	39	**3.16**	*0.23*	*0.01*	**2.11E-04**	**1.04E-22**	**8.53E-05**	AT1G42970	a,P	glyceraldehyde-3-phosphate dehydrogenase B (GAPB)
CATMA1a27050	40	**4.20**	*0.29*	*0.03*	**6.60E-07**	**4.24E-22**	**7.68E-06**	AT1G29070	P	ribosomal protein L34 family protein
CATMA1a64335	34	**4.00**	*0.29*	*0.03*	**8.82E-06**	**1.17E-20**	**1.03E-05**	AT1G74970	P	ribosomal protein S9 (RPS9)
CATMA5a36620	34	**4.23**	*0.32*	*0.03*	**3.06E-05**	**3.96E-21**	**7.34E-06**	AT5G40950	P	50S ribosomal protein L27, chloroplast (RPL27)
CATMA5a39880	34	**3.01**	*0.23*	*0.07*	**8.93E-05**	**1.17E-15**	**1.19E-07**	AT5G44130	w,p	fasciclin-like arabinogalactan-protein
CATMA1a13350	28	**3.28**	*0.26*	*0.01*	**1.31E-05**	**3.61E-19**	**6.76E-05**	AT1G14345	P	expressed protein, one transmembrane domain
CATMA3a11290	38	**2.98**	*0.25*	*0.02*	**2.46E-05**	**5.34E-22**	**6.59E-05**	AT3G12340	P	immunophilin-related
CATMA4a01510	23	**2.96**	*0.25*	*0.06*	**2.93E-05**	**1.70E-18**	**1.06E-03**	AT4G01310	c,P	ribosomal protein L5 family protein
CATMA4a23520	29	**3.77**	*0.32*	*0.05*	5.33E-02	**1.68E-12**	**8.70E-05**	AT4G21860	P	methionine sulfoxide reductase domain protein
CATMA4a26465	34	**4.05**	*0.35*	*0.05*	**2.79E-05**	**7.36E-18**	**4.05E-04**	AT4G24770	P	31 kDa ribonucleoprotein
CATMA1a60820	32	**3.12**	*0.27*	*0.03*	**2.10E-02**	**3.18E-19**	**8.43E-05**	AT1G71500	P	Rieske (2Fe-2S) domain-containing protein
CATMA4a17230	37	**3.28**	*0.30*	*0.03*	**5.81E-05**	**1.22E-19**	**6.59E-05**	AT4G16410	—	expressed protein
CATMA2a00475	32	**3.43**	*0.35*	*0.20*	**1.16E-04**	**1.64E-10**	**3.85E-05**	AT2G01420	—	auxin transport protein
CATMA4a21620	29	**2.96**	*0.30*	*0.06*	**3.23E-04**	**2.54E-19**	**8.87E-05**	AT4G20360	a,n,P	elongation factor Tu (TUFA)
CATMA1a00590	36	**2.96**	*0.32*	*0.11*	**5.48E-05**	**1.14E-15**	**1.85E-07**	AT1G01610	—	glycerol-3-phosphate acyltransferase
CATMA1a67690	35	**2.73**	*0.29*	*0.04*	**2.94E-03**	**1.49E-20**	**8.53E-05**	AT1G78630	P	ribosomal protein L13 family protein
CATMA1a67475	33	**3.48**	*0.39*	*0.23*	**1.49E-05**	**5.10E-08**	**3.19E-04**	AT1G78380	c,P,p,v	glutathione S-transferase, putative
CATMA2a30900	35	**2.63**	*0.31*	*0.08*	**1.43E-05**	**2.00E-17**	**1.10E-02**	AT2G32180	P	expressed protein
CATMA3a20410	34	**2.69**	*0.32*	*0.10*	**8.23E-06**	**8.91E-16**	**8.79E-05**	AT3G20680	P	expressed protein
CATMA5a19250	39	**2.55**	*0.32*	*0.17*	**3.50E-04**	**4.39E-14**	**8.94E-05**	AT5G20720	a,M,P	20 kDa chaperonin (CPN21)
CATMA3a52950	31	**2.72**	*0.36*	*0.21*	**4.03E-05**	**7.38E-12**	**6.45E-05**	AT3G59940	—	kelch repeat-containing F-box family protein
CATMA1a30400	31	**2.66**	*0.36*	*0.01*	**5.00E-05**	**1.33E-23**	**5.57E-03**	AT1G32060	a,P	phosphoribulokinase (PRK)
CATMA5a43410	23	*0.00*	**17.15**	**278.19**	**4.95E-09**	**2.53E-13**	**0.046445**	AT5G47450	v	major intrinsic family protein
CATMA3a46940	19	*0.02*	**10.04**	**116.88**	**7.21E-06**	**6.92E-12**	**0.024062**	AT3G53980	e	lipid transfer protein (LTP) family protein
CATMA3a00185	23	*0.08*	**12.14**	**141.58**	**1.49E-08**	**8.53E-21**	**6.09E-06**	AT3G01190	e	peroxidase 27 (PER27)
CATMA3a23750	16	*0.11*	**25.69**	**233.14**	**1.38E-05**	**4.82E-16**	**0.00675**	AT3G23800	—	selenium-binding family protein
CATMA1a40940	11	*0.14*	**17.74**	**11.32**	**2.3E-05**	**1.52E-05**	**0.011573**	AT1G49860	c	glutathione S-transferase
CATMA1a28300	19	*0.24*	**4.60**	**11.59**	**5.14E-05**	**2.23E-08**	**0.021175**	AT1G30270	c,n,p	CBL-interacting protein kinase 23 (CIPK23)
CATMA5a08890	20	*0.24*	**32.72**	**56.89**	**2.23E-10**	**6.53E-18**	**2.93E-05**	AT5G10130	e,x	pollen Ole e 1 allergen and extensin family protein
CATMA2a25985	17	*0.26*	**11.72**	**28.97**	**5.25E-06**	**7.78E-13**	**0.473892**	AT2G27550	—	centroradialis protein (CEN)
CATMA1a22610	22	*0.28*	**36.53**	**105.82**	**7.83E-06**	**4.44E-15**	**0.000233**	AT1G23720	w	proline-rich extensin-like family protein
CATMA3a23170	25	*0.29*	**13.03**	**75.65**	**7.07E-07**	**3.96E-21**	**1.58E-06**	AT3G23175	E	lesion inducing protein-related
CATMA1a61170	22	*0.30*	**2.78**	**3.42**	**8.77E-07**	**1.88E-07**	**0.468587**	AT1G71960	p	ABC transporter family protein
CATMA2a37070	24	*0.31*	**11.75**	**27.66**	**7.75E-05**	**3.99E-13**	**0.000278**	AT2G38800	—	calmodulin-binding protein-related
CATMA1a44600	16	*0.31*	**4.16**	**4.65**	**4.64E-05**	**1.63E-05**	**0.014032**	AT1G53590	v	C2 domain-containing protein
CATMA4a10345	14	*0.34*	**17.87**	**38.52**	**3.41E-06**	**2.36E-10**	**0.071489**	AT4G10310	p	sodium transporter (HKT1)
CATMA5a06070	27	*0.34*	**2.56**	**3.11**	**3.47E-08**	**1.77E-11**	**0.307964**	AT5G06850	—	C2 domain-containing protein
CATMA1a04113	20	*0.35*	**2.71**	**21.16**	**2.8E-05**	**8.54E-17**	**0.079992**	AT1G05260	E	peroxidase 3 (PER3)
CATMA1a28520	23	*0.36*	**5.05**	**5.28**	**3.41E-06**	**4E-11**	**2.69E-05**	AT1G30510	P	ferredoxin--NADP(+) reductase
CATMA3a48230	23	*0.37*	**7.91**	**20.28**	**2.97E-06**	**5.49E-15**	**0.027924**	AT3G55230	e	disease resistance-responsive family protein
CATMA5a09320	11	*0.37*	**5.73**	**13.92**	**4.28E-08**	**1.53E-17**	**2.74E-05**	AT5G10580	e	expressed protein

The table shows a list of genes, which have both a more shoot-like transcript level in long-term cytokinin-treated roots (Additional file 4: Table S3) and a more root-like transcript level in cytokinin-deficient shoots (Additional file 8: Table S4). Expression ratios are given for long-term cytokinin-treated roots and for cytokinin-deficient shoots in comparison to the mock-treated wild-type samples, as well as for the difference between roots and shoots. The expression ratios are coded: by different font variants: bold, up-regulation; italic, downregulation, regular, no regulation. The FDR-corrected p-values for the cytokinin effect indicate whether a gene was significantly regulated by cytokinin. The FDR-corrected p-values for the organ effect indicate whether the expression level of a gene was significantly different in roots and shoots. The FDR-corrected p-value for the interaction effect indicates whether cytokinin-dependent gene regulation was dependent on the organ. FDR-corrected p-values ≤ 0.03 were regarded as significant and marked with bold numbers. CATMA ID, unique identifier for the gene specific tag (probe) on the microarray (data can be queried under http://www.catma.org); AbB, number of spots above background (a rough measure for expression strength); AGI, unique gene identifier assigned by TAIR; Description, edited TAIR database annotation. Abbreviations for the subcellular localization are: a, apoplast; c, cytosol; E, endoplasmatic reticulum; e, endomembrane system; M, mitochondrium; n, nucleus; P, plastid; p, plasma membrane; v, vacuole; w, cell wall; x, extracellular space; —, not available or unknown.

The fact that cytokinin deficiency results in downregulation of chloroplast genes in green tissue, whereas excess cytokinin causes chloroplast genes expressed in the root is consistent with a primary role of cytokinin in regulating plastid development [41]. It is noteworthy that only a fraction (3.5%) of the ca. 3,000 nuclear genes encoding plastidic proteins [42] were affected by an altered cytokinin status, suggesting that cytokinin acts only on part of the genes and/or that there is a sequential order of cytokinin-regulated gene expression of which we detected only a part at the analyzed time point. Consistent with our data others found also a strong increase of genes encoding plastid proteins in cytokinin-treated root callus tissue, albeit at much later time points [10,43]. The expression of a comparatively small number of genes was shifted towards a more shoot-like profile in cytokinin-deficient roots (Figure 2), and correspondingly no shift of the cytokinin-deficient root sample towards the shoot coordinates was observed in the PCA (Figure 1b-e).

Taken together, these findings indicate that the cytokinin status apparently determines a more root- or shoot-like transcriptomic pattern that is at least partially independent of morphologically discernable altered organ identity. We used the category of nuclear genes encoding plastid proteins as a diagnostic tool to assess the “shootyness” of the transcriptome but it is impossible to determine whether the transcriptomic shift in “shootyness” is cause or consequence of a shift in organ identity. However, several studies dissecting the temporal pattern of gene expression during cytokinin-induced shoot development from root calli found that shoot marker genes start to be upregulated only several days after the onset of the hormonal treatment [10,44-46] supporting the notion that during the time period analyzed here no shoot identity was obtained by the cytokinin-treated root tissue.

### Similarity of the overall transcriptional response of roots and shoots to cytokinin

The effect of cytokinin treatment at different time points was not resolved in the PCA analysis, because the large organ and genotype effects prevailed. Therefore, we removed the largest effect — the organ effect — by normalizing the root and shoot samples for each experimental condition as described in Methods. As a result, support tree clustering showed distinct root-shoot-pairs at each experimental condition indicating that the overall transcriptional response to cytokinin is similar in roots and shoots (Figure 1f). The two early time points of cytokinin induction showed the closest relation, while the long-term treated samples separated from the other wild-type samples.

PCA analysis using this dataset normalized for the organ effect resolved two temporal phases of the transcriptomic response to cytokinin. For the early time points of cytokinin induction, the separation is approximately parallel to the z-axis and for the later time points parallel to the y-axis, suggesting that the late response affects a different set of genes than the early response (Figure 1j). Both root and shoot samples follow a similar pattern (shown as dashed lines in Figure 1j), indicating that the transcriptomic response to cytokinin treatment is similar in both organs. This conclusion was confirmed by an additional PCA without the cytokinin-deficient samples, where the arrangement of the wild-type samples in one plane remained stable (data not shown).

Given the fundamental differences of the growth response of roots and shoots to cytokinin, we initially hypothesized that this could be reflected by large differences at the transcriptome level. In fact, cytokinin response genes are regulated by the hormone in a context-dependent manner [47], which is likely due to the presence of multiple *cis*-regulatory elements in their promoters. Indeed, the only known cytokinin response element is not present in the promoters of all cytokinin responsive genes [19], suggesting that a variety of possibilities exist to regulate a gene by cytokinin. It might well have been possible that large specific subsets of genes are regulated by cytokinin in an organ-specific manner. However, that appears not to be the case. Therefore it is remarkable and to some extent unexpected that the global transcriptional responses in both organs appear to be rather similar. This indicates that it is not the differential bulk expression determining the rather different cytokinin-dependent growth responses in both organs. This would also imply that cytokinin signaling to the nucleus is to a large extent similar and that organ-specific features are limited to few changes, which regulate the growth response. It would be very interesting to find out how these comparably small differences can drive completely different growth responses in roots and shoots. Furthermore, because of these relatively small differences one can expect a relatively low number of genes that are regulated by cytokinin in an organ-specific manner.

It should be noted that the absolute expression level of the genes, although showing qualitatively similar cytokinin responses, can be quite different in roots and shoots (Additional file 2: Table S1 and Additional file 3: Table S2). For instance, the transcripts encoding the auxin transporter PIN2 or the tonoplast intrinsic protein TIP2;3 are more than hundred-fold more abundant in the root than in the shoot, while the phosphate transporter gene *PHT2;1* shows a much higher expression level in the shoot (Additional file 6: Figure S3, Additional file 7: Figure S4). Thus the differential response of the tissues to cytokinin may be affected by additional factors that regulate the absolute abundances of the different messages.

### Chronic cytokinin deficiency and cytokinin induction affect different sets of genes

The PCA of the normalized dataset (Figure 1g-j) shows that the CKX samples separate from the wild-type samples along the x-axis while the effects of cytokinin treatment on the transcriptome are distributed over the y- and z-axes and remain within the same plane (Figure 1g-j, purple markings). This means that the transcriptomic changes resulting from chronic cytokinin deficiency are not simply the opposite of those resulting from early or late cytokinin treatment, but that another (third) set of genes is affected. Consequently, we analyzed cytokinin-deficiency separately from the dataset derived from the cytokinin-induced plants.

### Regulation of cytokinin metabolism and signalling genes

**Figure 3:**
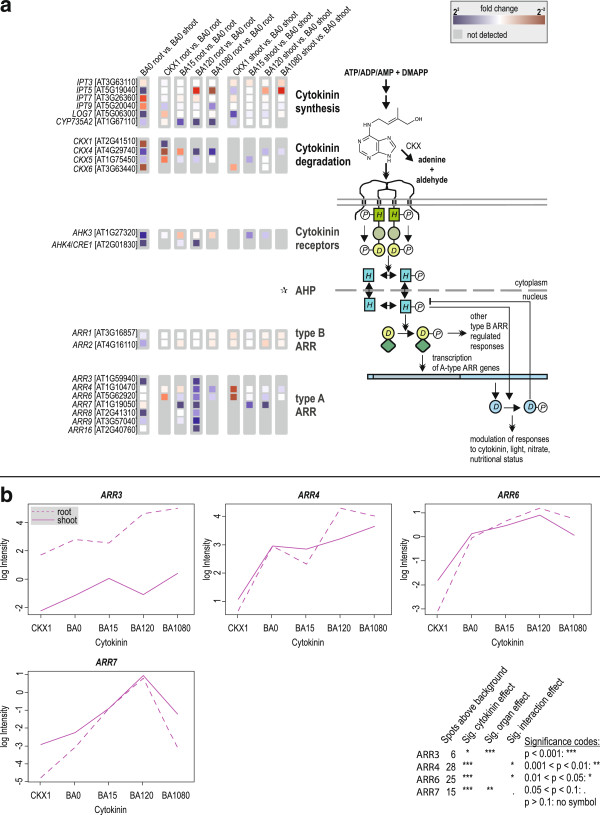
**Differential expression of cytokinin signalling and related genes in root and shoot in response to the cytokinin status.** (**a**) Changes in expression of cytokinin-related genes evaluated with Mapman [157]. Genes associated with functions in cytokinin metabolism or signalling are listed on the left side, and a schematic drawing of cytokinin signal transduction is shown on the right side. Colour coding of the boxes in the middle shows changes of transcript abundance between the samples indicated at the top. Blue indicates an upregulation, red a downregulation. Grey boxes denote genes, which are detected in less than two out of four arrays of the higher expressed condition. Cytokinin-related genes which were never detected or not present on the microarray are not shown. The colour codes were generated using the Mapman software and aligned to a figure using standard image processing software. : None of the *Arabidopsis* histidine phosphotransmitter genes is present on the CATMA V2.3 microarray. (**b**) Interaction plots and significance of changes of transcript abundance of selected A-type *ARR* genes. The interaction plots show the relative fluorescence intensity (in log scale) of the spots on the array for the gene indicated on top of each graph in the cytokinin-deficient and cytokinin-treated samples for roots and shoots separately.

To search for potential homeostatic mechanisms within the cytokinin system, we studied the influence of cytokinin treatment and cytokinin deficiency on the transcript level of cytokinin metabolism and signalling genes (Figure 3). Unfortunately, the CATMA microarray lacks a part of these cytokinin-related genes and expression of another part was below the detection limit. Therefore, only a limited number of the relevant genes could be analyzed. A subset of these showed significant differences in organ specificity and cytokinin response of transcript abundance.

Among the cytokinin-synthesizing *IPT* genes, *IPT5* showed a higher expression in the shoot under all conditions, while *IPT7* (and less strongly also *IPT9*) was consistently more highly expressed in the root (Figure 3a). *IPT5* was strongly downregulated in long-term cytokinin-treated roots and upregulated in cytokinin-deficient shoots (see also Figure 3a). In contrast, a strong upregulation in long-term cytokinin-treated roots was detected for *IPT9,* and *IPT3* was apparently neither differentially expressed nor regulated by cytokinin. The gene encoding the cytokinin hydroxylase CYP735A2 was strongly upregulated by cytokinin in a root-specific fashion (see below). Among the cytokinin-degrading *CKX* genes, strong differences were found for *CKX4* and *CKX5*, confirming previous data [48]. Both genes were expressed more strongly in the root than in the shoot and both were upregulated by cytokinin and downregulated under conditions of cytokinin deficiency, the response in the root being stronger than in the shoot (Figure 3a). *CKX4* has been consistently described as a primary target gene of B-type response regulator ARR1 [49]. In contrast to these two genes, *CKX6* was expressed more strongly in the shoot and was hardly regulated by the cytokinin status in our study. Please note that the strong upregulation of *CKX1* found in cytokinin-deficient roots results from detection of the transgene. Of the putative cytokinin transporter genes, *PUP4* and *PUP11*[50] were more strongly expressed in the root and both showed a transient upregulation in the shoot but not in the root in response to cytokinin (Figure 3a).

The known higher expression of the cytokinin receptor gene *CRE1/AHK4* in the root [51,52] and its induction by cytokinin [53] was confirmed. The transcript level of the two B-type *Arabidopsis* response regulator genes (*ARR1*, *ARR2*) were more strongly expressed in the root and – as expected – not regulated by cytokinin (Figure 3a). In contrast, all known primary cytokinin response genes encoding A-type response regulators (*ARR4*, *ARR6*, *ARR7*, *ARR9*, and *ARR16*) detected were equally responsive to cytokinin in both roots and shoots (Figure 3a, Additional file 6: Figure S3a, Additional file 7: Figure S4a, b). Figure 3b shows the reduced expression levels of four exemplary A-type *ARR* genes (*ARR3*, *ARR4*, *ARR6*, and *ARR7*) in *35S:CKX1* transgenic roots and shoots as compared to the corresponding wild-type tissues and their induction pattern following cytokinin treatment in roots and shoots of the wild type (see also Additional file 6: Figure S3c and Additional file 7: Figure S4c, d). Despite a generally similar response to an altered cytokinin status, the kinetics and amplitudes of changes in steady state transcript levels differ for the four genes.

Together, these data confirm that the transcript abundance of several cytokinin metabolism and signalling genes depends on the cytokinin status [6,9,13,15,17] indicating regulatory loops between cytokinin metabolism and signalling. The analysis also showed that the reaction patterns are not uniform but complex, with differences in kinetics and organ specificity.

### Known and novel genes responding to cytokinin

In the following sections we will analyse separately the sets of genes that show altered expression patterns following either a treatment of seedlings with cytokinin or under conditions of constitutive cytokinin deficiency (Additional file 2: Table S1 and Additional file 3: Table S2). The organ-specific regulation of some known cytokinin response genes will be addressed and an arbitrary selection of novel cytokinin-regulated genes, which may have escaped previous discovery due to unspecific sampling, is discussed. We will explore similar and differential changes of steady-state transcript levels in both organs, as well as changes that are restricted to either roots or shoots. This organizing principle is followed, although in part genes belonging to a similar context (e.g. related to auxin or brassinosteroid, meristem regulation, nutrient transport, etc.) were categorized into different groups of organ-specific cytokinin-regulation.

### Cytokinin-induced changes of transcript abundance

**Figure 4:**
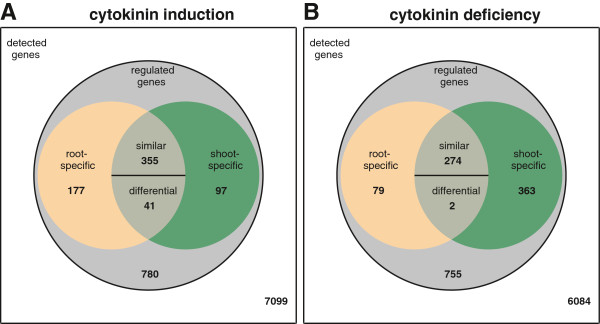
**Venn diagrams showing different organ-specific classes of cytokinin-regulated genes.** (**A**) The Venn diagram shows the numbers of detected and regulated genes following 18 h of cytokinin treatment. (**B**) The Venn diagram shows the numbers of detected and regulated genes in cytokinin-deficient seedlings. The gene classification was performed as described in Methods and Additional file 6: Figure S3.

1,450 of the 8,549 genes (i.e. ~17%) that were detected by the arrays were responsive to a short-term or long-term cytokinin treatment (Figure 4). Out of these, 670 genes could be assigned unambiguously to one specific category as follows: the majority, i.e. 396 genes, were regulated in both organs with either a similar or differential expression pattern. The former genes (355) were regulated in both organs in the same direction, while the latter genes (41) were regulated in both organs in the opposite direction. 177 genes were regulated by cytokinin in a root-specific fashion, and 97 genes were regulated in a shoot-specific fashion. A large number of genes (780) were regulated significantly by cytokinin under at least one of the experimental conditions, but lacked a significant organ effect. Most of these genes showed a different steady-state transcript level at only one time point (Additional file 2: Table S1). They were not further analysed, as the focus of this work was on organ specificity. 7099 genes were classified as non-regulated under our conditions (Figure 4 and Additional file 2: Table S1f).

### Assignment of organ-specificity to known cytokinin response genes

Firstly, we examined whether we could assign a root- or shoot-specific response to known cytokinin-regulated genes which had been discovered previously by genome-wide microarray analyses [6,9,13,15,17]. In fact, a large number of these genes were detected by the CATMA array and most, but not all, of them were found to be regulated in both organs (Table 4 and Additional file 9: Table S5).

**Table 4:** **Changes of transcript abundance of known cytokinin-regulated genes of *Arabidopsis thaliana***

**Category**	**AGI**	**Description**	**Ratio**								**Significance code**		**Previously published in**
			**Root**				**Shoot**						
			**CKX1 vs. BA0**	**BA15 vs. BA0**	**BA120 vs. BA0**	**BA1080 vs. BA0**	**CKX1 vs. BA0**	**BA15 vs. BA0**	**BA120 vs. BA0**	**BA1080 vs. BA0**	**Cytokinin effect**	**Interaction effect**	
root-specific	AT2G40230	transferase family protein	1.07	0.81	**2.72**	**2.99**	2.32	0.93	1.18	2.13	**	·	A B C E
	AT1G67110	cytochrome P450, cytokinin hydroxylase (CYP735A2)	0.61	**4.20**	**60.65**	**29.72**	2.09	1.05	0.90	2.06	***	***	A C E F
	AT2G01830	histidine kinase CRE1/AHK4	0.64	1.39	**4.14**	0.77	1.60	1.38	0.83	1.92		*	A C F
	AT4G16990	disease resistance protein TIR-NBS-LRR class	*0.13*	0.60	0.47	**2.63**	0.87	0.57	1.02	0.88	*	*	A F
	AT4G19170	9-cis-epoxycarotenoid dioxygenase	0.93	*0.3*	*0.18*	1.08	1.55	0.68	0.59	0.64	**		A F
	AT1G17190	glutathione S-transferase	1.77	**2.85**	**7.63**	1.07	1.75	1.20	0.77	0.56	*	*	C F
	AT5G47980	transferase family protein	*0.35*	1.61	**6.18**	**14.36**	0.82	0.47	0.56	2.00	**	*	C F
shoot-specific	AT3G48750	A-type cyclin-dependent kinase	0.95	1.00	1.40	0.65	**4.01**	**8.78**	**7.92**	1.84	·		A E
	AT3G62930	glutaredoxin family protein	1.97	2.10	2.06	2.10	*0.36*	0.88	1.58	2.03	*	·	C E
	AT3G21670	nitrate transporter NTP3	0.84	0.70	0.72	1.36	2.00	**2.66**	**2.65**	**4.47**	*		C F
	AT5G53290	ERF/AP2 subfamily B-5 member (CRF3)	1.00	2.28	1.71	0.79	**3.03**	**5.47**	**7.94**	**6.26**	***	***	D
in both organs	AT1G69530	expansin At-EXP1	0.89	1.52	2.18	**13.23**	0.93	1.00	1.27	**4.16**	***		A B C E
	AT4G29740	cytokinin oxidase CKX4	*0.09*	0.43	**4.30**	**2.88**	0.83	1.20	1.90	**4.44**	***	**	A C E F
	AT1G19050	A-type response regulator ARR7	*0.31*	**4.53**	**14.48**	0.97	0.63	**2.57**	**9.23**	2.07	***		A B F
	AT2G40670	A-type response regulator ARR16	0.75	**2.73**	**12.36**	1.58	2.04	0.82	**4.93**	**5.68**	*		A B F
	AT2G01830	histidine kinase (CRE1/AHK4)	0.64	1.39	**4.14**	0.77	1.60	1.38	0.83	1.92		*	A C F
	AT1G10470	A-type response regulator ARR4	*0.21*	0.64	**2.53**	2.10	*0.27*	0.93	1.20	1.61	***		A B
	AT2G38750	annexin	*0.09*	1.73	**10.8**	**6.06**	1.15	1.80	**2.53**	0.69	***	**	A C
	AT5G64620	invertase/pectin methylesterase inhibitor family protein	1.54	0.67	0.71	*0.38*	*0.11*	*0.37*	*0.30*	0.47	**	***	A C
	AT2G20520	fasciclin-like arabinogalactan-protein FLA6	1.65	0.54	1.97	**6.21**	**4.24**	1.18	**2.64**	1.00	·	·	A E
	AT2G35980	harpin-induced family protein YLS9	*0.03*	1.67	**4.25**	**5.87**	**3.26**	**4.39**	1.02	**5.26**	***	***	A F
	AT4G10120	sucrose-phosphate synthase, putative	**4.24**	1.20	0.41	**5.24**	**2.76**	1.38	1.87	0.71	·	·	A F
	AT4G27410	no apical meristem (NAM) family protein RD26	0.78	0.60	1.68	**2.62**	0.44	0.70	0.85	0.82	*		B F
	AT1G13420	sulfotransferase family protein	0.48	0.68	**15.48**	1.41	**5.43**	1.82	0.59	1.00		**	C F
	AT3G50300	transferase family protein	1.92	1.91	**5.25**	**3.00**	2.38	*0.12*	*0.28*	1.45		·	C F
	AT3G57010	strictosidine synthase family protein	1.24	1.41	**7.99**	**4.19**	**9.13**	2.47	**4.27**	**7.07**	***	*	C F
	AT5G42590	cytochrome P450 CYP71A16	0.76	0.77	**11.73**	1.74	1.14	*0.36*	*0.38*	0.54	**	***	C F
	AT5G51440	mitochondrial small heat shock protein HSP23.5-M	**15.18**	**3.34**	**3.32**	**5.58**	0.88	1.42	**14.16**	2.29	*	*	C F
	AT1G75820	CLAVATA1 receptor kinase (CLV1)	**2.63**	1.05	0.79	0.54	0.42	*0.34*	*0.32*	*0.09*	***	***	A
	AT5G57090	auxin transport protein (EIR1)	**3.66**	2.12	0.89	0.59	**35.26**	1.36	1.15	0.86	***	*	A
	AT5G62920	A-type response regulator ARR6	*0.12*	1.62	**2.35**	1.73	*0.26*	1.28	1.72	0.96	***	·	B
	AT3g61630	ERF/AP2 member (CRF6)	1.59	0.93	0.97	**3.37**	*0.27*	0.84	0.65	1.31	***	***	D

A selection of previously published cytokinin-responsive genes from various publications reporting changes within 24 h were analyzed for their changes in transcript abundance in response to cytokinin induction or cytokinin deficiency in root and shoot samples. Expression ratios are coded by font-variants as described for Table 1. Significance codes indicate p-values calculated as described in Methods: 0.1 > · > 0.05 > * 0.01 > ** 0.001 > ***. Genes that were published in at least two previous publications and selected examples of known regulated genes that were only published once are listed here. In addition, selected examples of known regulated genes that were only published once were added. A more comprehensive list of genes can be found in Additional file 9: Table S5. The publications are coded by letters: A, Rashotte et al., 2003 [15]; B, Brenner et al., 2005 [9]; C, Kiba et al., 2005 [13]; D, Rashotte et al., 2006 [16]; E, Taniguchi et al., 2007 [17]; F, Argyros et al., 2008 [6]. AGI, unique gene identifier assigned to *Arabidopsis thaliana* genes by TAIR.

A noteworthy example of a root-specific cytokinin response gene encodes the cytokinin hydroxylase CYP735A2 which responded strongly to cytokinin within minutes. Its transcript level in roots after 120 min of cytokinin treatment was strongly increased, while it was non-responsive in shoot tissue (Table 4, Additional file 6: Figure S3a). Significantly, the basal level of *CYP735A2* expression was similar in both roots and shoots (Additional file 6: Figure S3b, Additional file 7: Figure S4a, b). This regulation is interesting because the hydroxylase catalyzes the conversion of isopentenyl riboside phosphates to *trans*-zeatin (*t*Z) riboside phosphates [54], which are inactive precursors of *t*Z-type cytokinins. *t*Z-type cytokinins are the main cytokinins transported in the xylem from roots to shoots [5,55]. The exclusive induction of *CYP735A2* by cytokinin in roots, which probably results in a locally increased production of *t*Z-type cytokinins, lends support to *t*Z-type cytokinins having a distinct role in root-to-shoot communication [55-57].

An example of a shoot-specific regulated known cytokinin response gene is *CDKA;1*, encoding a cyclin-dependent kinase gene. CDKA;1 is an important cell cycle regulator and is involved in a variety of developmental processes. Because cells expressing more *CDKA;1* are more competent for cell division, it is thought to be a prerequisite for post-embryonic cell division [58,59]. It was shown recently that the CDK activity level in the shoot apical meristem is important for cellular differentiation [60], a process that is under cytokinin control [30-32]. *CDKA;1* regulation in the shoot by cytokinin could be part of the activities orchestrated by cytokinin in the shoot apical meristem.

The organ-specificity of some other known cytokinin response genes is also described in the following sections.

### Genes with a similar response in roots and shoots upon cytokinin treatment

Of the 355 genes displaying a similar cytokinin response in roots and shoots, 133 genes showed an increased transcript level following cytokinin treatment, while the transcript level of 222 genes was reduced (Additional file 2: Table S1). 66 genes showed a rapid change of transcript abundance within 15 min and thus belong to the immediate-early response genes. The vast majority of the changes became apparent 120 min or 1080 min after the treatment. Interestingly, the transcript level of many genes that was increased after 120 min remained increased also after 1080 min. As already mentioned above, it should be noted that there are substantial differences in the basal expression levels of numerous of these genes in roots and shoots which could be of functional relevance as well. An example is the *CLV1* gene discussed below, which is approx. 4-fold higher expressed in the untreated wild-type shoots compared to the roots (Additional file 6: Figure S3b, Additional file 7: Figure S4a, b).

Among the upregulated developmental genes, a particularly interesting one encodes the CLAVATA (CLV) formation protein SHEPHERD (SHD, AT4G24190). SHD is a member of the HSP90 protein family and has been suggested to act as a chaperone for the correct folding of nascent CLV1 and/or CLV3-like polypeptides in the endoplasmatic reticulum [61]. Consistently, *shd* mutant plants are phenotypically reminiscent of *clv* mutants. The CLV1 and CLV3 proteins are part of the regulatory loop controlling meristem size and activity in concert with WUSCHEL (WUS) [62]. Cytokinin regulates the expression of *CLV1* and *WUS*, thereby controlling meristematic activity [30,31,33]. Transcriptional regulation of the *SHD* gene possibly represents an additional activity of cytokinin in regulating meristematic activity.

Members of the ERF/AP2 family were noticeably frequent among the cytokinin-responsive transcription factor genes. The transcript levels of eight members of the family (AT5G07580, AT1G77640, AT5G61600, AT1G28370, AT3G15210, AT5G51190, AT5G47220, and AT2G44840) were strongly downregulated by the hormone, but none was upregulated (Table 1, Additional file 6: Figure S3a, Additional file 7: Figure S4a, b). This shows that, in addition to the ERF/AP2 subclass of cytokinin-regulated factors (CRF) with its current six members [63], other members of other subclades of the ERF/AP2 family are also cytokinin-regulated (see also below).

Interestingly, numerous ribosomal genes were identified as cytokinin responsive in this study as well as in previous studies [9,13,63]. Several developmental processes react sensitively to mutations in ribosomal genes, including embryogenesis, root growth and leaf development, underpinning the functional relevance of the specific assembly of ribosomal proteins in addition to their fundamental role in translation, possibly because they are required to translate specific proteins [64-66]. According to the Gene Ontology database of May 2011, the *Arabidopsis* genome encodes 474 ribosomal proteins [67,68] and at least 87 transcripts of them responded to cytokinin (Additional file 10: Table S6). The cytokinin-responsive ribosomal genes encode proteins of both plastidic and cytoplasmic localisation. Noteworthy, most of the transcripts of nuclear genes encoding plastidic ribosomal proteins reflect the organ shift discussed above, i.e. induction in long-term cytokinin-treated roots and downregulation in cytokinin-deficient shoots. Ribosomal genes encoding cytosolic proteins show diverse cytokinin response patterns. The altered composition of ribosomes and the translational changes that this may cause are a largely unexplored part of the cytokinin response.

The flavonol synthase gene *FLS1* was strongly upregulated, particularly in the shoot. *FLS1* is part of the flavonol biosynthesis pathway [69] and it is likely that its strong upregulation is part of the induction of anthocyanin accumulation by cytokinin [70]. Cytokinin and flavonoids are involved in regulating several developmental processes: both, for example, are positive regulators of nodule organogenesis [71] but have opposite roles in the control of shoot branching [72-74]. Flavonoids are inhibitors of auxin transport and thus may mediate crosstalk between cytokinin and auxin action. In any case, there are multiple links between cytokinin and flavonoids and effects on the *FLS1* transcript level could be part of that. *FLS1* was also found among the strongly upregulated genes in cytokinin-deficient plants (see below), illustrating nicely that cytokinin deficiency does not simply induce the opposite changes caused by cytokinin treatment, as already shown by the PCA discussed above.

Five members of the nodulin *MtN21* gene family are downregulated by cytokinin: AT2G28120, AT4G08290, AT4G01450, AT4G08300, and AT3G25190 (Additional file 6: Figure S3a, Additional file 7: Figure S4a, b). The role of nodulin MtN21-like genes is not yet understood, but the presence of seven transmembrane domains and structural homologies with bacterial multidrug exporters might suggest a role in a transport function. It was proposed that the maize nodulin MtN21-like gene is probably involved in the transport of a component related to vascular tissue assembly [75], which is interesting in view of the role of cytokinin in vascular development [76].

Several other regulated genes can be associated with known functions of cytokinin, such as crosstalk with other hormones including auxin (e.g. downregulation of *IAA7* and *IAA13*) and gibberellin (downregulation of the gibberellin 2-oxidase gene AT1G02400) or its functions in mineral uptake (e.g. *IRT2*, *ZIP4*) (Additional file 2: Table S1).

### Root-specific changes in gene expression upon cytokinin treatment

Among the 177 genes that were regulated only in roots, the strongest differences in expression level were detected for a gene encoding a member of the legume lectin family protein, its transcript abundance increased >150-fold (Additional file 2: Table S1). As has been discussed above, the largest specific group of regulated genes encoded plastid proteins, and a particularly interesting gene showing root-specific regulation was cytokinin hydroxylase gene *CYP735A2*. A root-specific regulation by cytokinin was assigned to two other cytochrome P450 genes for which cytokinin-regulation has been previously described [14,15]. One of them, AT2G34490, encodes the C22 sterol desaturase CYP710A2 involved in brassinosteroid metabolism. It catalyzes the final step in the biosynthesis of brassicasterol and stigmasterol [77,78]. This is interesting as cytokinin and brassinosteroids have been recently shown to act in common pathways in regulating root architecture [79]. The molecular basis for this crosstalk is not known but it is conceivable that regulation of metabolism genes may be part of it. Noticeably, the related gene encoding CYP710A1 and the brassinosteroid biosynthesis gene *DIM/DWF* are deregulated in cytokinin-deficient plants (see below) indicating that brassinosteroid metabolism is regulated by cytokinin on different levels.

The largest fold-change in transcript abundance between roots of the cytokinin-deficient and long-term cytokinin-treated seedlings was noted for the *LBD4* gene, encoding a member of the LOB domain protein family [80] and already noted before in relation to cytokinin [14]. The function of *LBD4* is unknown, but members of this plant-specific family of DNA-binding transcription factors are involved in various developmental processes, such as leaf polarity, and floral or inflorescence architecture in different plant species [80-84], as well as lateral root formation and the regulation of anthocyanin and nitrogen metabolism [85].

Again, a number of genes were identified that are relevant for auxin action, e.g. encoding the IAA-amido synthase DFL1 (AT5G54510) and PIN4 (AT2G01420), suggesting that cytokinin-auxin crosstalk is realized in many different ways in a tissue-specific fashion. Similarly, genes encoding proteins involved in mineral uptake are also regulated in a root-specific fashion, including iron transporter gene *IRT1* as well as phosphate (*PHT2;1*, AT3G26570) and sulfate transporter genes (Additional file 2: Table S1, Additional file 6: Figure S3a, Additional file 7: Figure S4a, b).

### Shoot-specific changes in gene expression upon cytokinin treatment

97 genes were exclusively regulated in the shoot, of which 23 were upregulated (up to 6-fold), and 74 were downregulated, in both cases most consistently after 120 and 1080 min. One of the most prominent genes that was rapidly and strongly downregulated in the shoot is *CLV1* (Table 1, Additional file 6: Figure S3a, Additional file 7: Figure S4a, b). Its negative regulation by cytokinin has been previously reported [30,31,33], but the shoot-specific nature of this was not known. Similarly, cytokinin regulation of *CRF3*, encoding a member of the subfamily B-5 of the ERF/AP2 transcription factor family (AT5G53290), had been shown before [16], but not its shoot-specificity (Table 1). Another member of the ERF/AP2 transcription factor family (subfamily B-3), *ERF5* (AT5G47230), is one of the most strongly downregulated genes in the shoot (Table 1). ERF5 is an activator of GCC box-dependent transcription and is also regulated by ethylene and abiotic stress [86].

The gene encoding the digalactosyldiacylglycerol synthase 1 (*DGD1*, AT3G11670) was upregulated almost 6-fold by cytokinin treatment. DGD1 catalyzes the synthesis of the galactolipid digalactosyldiacylglycerol (DGDG), requiring monogalactosyldiacylglycerol (MGDG) as a substrate, which is synthesized by mongalactosyldiacylglycerol synthase (MGD) [87,88]. Both galactolipids constitute the predominant and functionally relevant component of thylakoid membranes [89]. Previously, it was shown that the accumulation of MGDG in cucumber seedlings required cytokinin in addition to light, and that both factors play a co-operative role [90]. The response of *DGD1* shown here indicates that the cytokinin regulation also comprises this step of galactolipid synthesis, adding another example of the influence of the hormone on chloroplast function.

The serin/threonine protein kinase gene *PINOID (PID)*[91], encoding a regulator of the auxin response, is downregulated approximately 3-fold. PID phosphorylates the auxin efflux carrier proteins PIN, thereby changing their apical-basal polar localization and thus increasing basipetal polar auxin transport [92-96]. Although it is not known whether transcriptional regulation of *PID* plays a role in regulating its kinase activity, it is possible that the downregulation of *PID* by cytokinin causes an increase in basipetal auxin transport. In any case, in whatever functional context, regulation of *PID* by cytokinin could be a mechanism in addition to the known regulation of *PIN* genes [29,97,98] that contributes to the manifold regulatory influences of cytokinin on auxin function.

### Genes with differential expression patterns in root and shoot upon cytokinin treatment

41 genes were upregulated in shoots and downregulated in roots or vice versa*.* The existence of such a differential organ-specific response pattern could be, for example, achieved by transcription factors that act depending on the cellular or developmental context either as activator or repressor [99]. However, given the low number of genes in this class and the frequently transient nature of their differential regulation, this does not seem to be a general feature of differential cytokinin activity in roots and shoots. Two genes encoding cytochrome P450 enzymes belong in this class and show altered transcript levels at several time points (Additional file 2: Table S1). One of these genes encodes CYP83A1, also called REF2 (AT4G13770), catalyzing the initial conversion of aldoximes to thiohydroximates in the synthesis of glucosinolates not derived from tryptophan. It has also a role in auxin homeostasis [100-102]. *F5H2* (AT5G04330) encoding a ferulate 5-hydroxylase involved in lignification [103] is regulated in the opposite way, namely up in the root and down in the shoot. The functional significance of this type of regulation is not known.

### Genes differentially expressed under conditions of constitutive cytokinin deficiency

In cytokinin-deficient tissues of *35S:CKX1* plants, 7,557 genes were detected, of which 1,473 (~20%) showed an altered transcript abundance compared to the wild type. 718 of these genes could be assigned to one specific category. The major effect of cytokinin deficiency was manifested in the shoot, where 639 genes were differently expressed. 363 of these genes were exclusively affected in this organ and 277 were affected in the root as well. A smaller number of 79 genes were regulated only in the root and 2 genes were identified showing differential regulation in root and shoot (Additional file 3: Table S2, Figure 4). One outcome of the analysis was the relatively small overlap of genes affected by cytokinin-deficiency or cytokinin-treatment as described above. There are examples of opposite gene regulation in both conditions, e.g. the A-type *ARR* genes, which are upregulated by cytokinin and downregulated under cytokinin deficiency. Conversely, there are several cases of a change of transcript level in the same direction under both conditions (e.g. *FLS1*, several *AP2/ERF* transcription factor genes) illustrating that opposite hormonal conditions may have a similar effect on transcript abundance. The relative shift of the shoot expression pattern towards a more root-like pattern, especially concerning genes encoding plastid proteins, was particularly noteworthy in the light of the effects of long-term cytokinin treatment on the roots described above. In addition, the data show that roots and shoots clearly react with different trancriptomic changes to cytokinin deficiency. Exceptions are general indicators of the cytokinin status, such as the A-type *ARR* genes, which are lowered in both organs (Figure 3). The alteration of the transcript level of several regulated genes may contribute to the establishment of the cytokinin deficiency syndrome.

### Genes with similar reaction in root and shoot under constitutive cytokinin deficiency

275 genes showed a similar response in roots and shoots: 113 thereof were upregulated and 162 were downregulated (Additional file 3: Table S2). There are several regulated genes that provide insight into the biological functions of cytokinin, such as the strong downregulation of nitrate reductase genes *NR1* and *NR2* reflecting the functional link between cytokinin and nitrogen usage [104].

A very strongly altered expression level was shown by the sodium transporter gene *HKT1*, which was upregulated 24-fold in roots and 16-fold in shoots (Additional file 3: Table S2, Additional file 6: Figure S3c, Additional file 7: Figure S4c, d). The main function of HKT1 is the control of Na^+^ entry into plant roots and the root/shoot distribution of Na^+^[105,106]. Thus, HKT1 has an important role in mediating salt tolerance, as has been shown in ecotypes as well as by forward genetics, and it has been utilized to generate salt-tolerance using a transgenic approach [107-110]. The regulation found here is consistent with the previous finding that *HKT1;1* is repressed by cytokinin treatment but showed significantly elevated expression in the B-type response regulator double mutant *arr1 arr12*[111]. These data suggested that cytokinin, acting through the transcription factors ARR1 and ARR12, regulates sodium accumulation in the shoots by controlling the expression of *HKT1* in the roots. Cytokinin receptors AHK2 and AHK3 were consistently identified as negative regulators of the osmotic stress response and receptor mutants as well as mutants with a reduced cytokinin content were found to be salt resistant [112,113]. Another cytokinin-regulated transporter gene was *ZIP1* (AT3G12750), which encodes a Zn^2+^ transporter and was upregulated 3.2- and 4.4-fold in shoots and roots, respectively [114]. Its upregulation by cytokinin deficiency is in accord with the finding that cytokinin-deficient plants accumulate higher amounts of Zn^2+^ in their shoot tissue [26].

Among the transcription factor genes, four *ERF/AP2* transcription factor genes (AT1G28370, AT3G16770, AT5G05410, and AT3G15210) are downregulated to about one third of their expression level (Additional file 6: Figure S3c, Additional file 7: Figure S4c, d). Interestingly, three of them are identical to the cytokinin-regulated AP2/ERFs mentioned above (see Table 1), indicating again that increase and decrease of the cytokinin status may have similar consequences.

*MYB12* is another strongly regulated transcription factor gene. MYB12 belongs to subgroup 7 of the R2R3-MYB family and is a flavonol-specific activator of flavonoid biosynthesis [115]. Together with its two other subgroup members, MYB11 and MYB111, it strongly activates the promoters of genes encoding chalcone synthase (CHS), flavanone 3-hydroxylase (F3H), flavonol synthase (FLS1), and – to a lesser extent – chalcone flavanone isomerase (CHI), which are all involved in the formation of flavonols [116]. The upregulation of *MYB12* consistently resulted in an enhanced transcript level of *CHS* (AT5G13930) and *FLS1* (Additional file 3: Table S2). We hypothesize that cytokinin-deficient plants contain higher levels of flavonols and/or flavonoids, which have a variety of functions in plant stress defence. Interestingly, auxin and ethylene induce flavonol accumulation through partly identical transcriptional networks [117].

Two well known auxin-related genes, *PIN2* and *AXR1*, were upregulated under cytokinin-deficiency. *PIN2* belongs to those genes which show a much higher shoot expression in response to cytokinin deficiency (55-fold increase compared to wild-type shoots) while it is normally predominantly expressed in roots (Table 2 and Additional file 3: Table S2, Additional file 6: Figure S3c, d, Additional file 7: Figure S4, Additional file 6: Figure S3, d). *PIN2* encodes a member of the auxin efflux carrier proteins [118-121]. Its regulation by cytokinin has been reported in the context of root meristem size regulation [97,122], but it might also be involved in co-ordinating the activities of cytokinin and auxin in regulating root gravitropism [123]. *AXR1*, which was upregulated about 5-fold in both organs, encodes part of a dimeric E1 ubiquitin-activating enzyme of the ubiquitin-proteasome protein degradation pathway [124-127] mainly involved in regulating the auxin response, but also other signalling pathways dependent on ubiquitin-dependent protein degradation [128-133]. The upregulation of *AXR1* may enhance the potential for neddylation of proteins to be targeted for degradation by the proteasome. The Aux/IAA proteins, transcriptional repressors of auxin-inducible genes, are primary targets of this pathway. Higher expression of *AXR1* under cytokinin-deficiency suggests an enhanced auxin signalling in cytokinin-deficient plants. The identification of *AXR1* as a regulatory target of cytokinin adds another potential regulatory level to cytokinin-auxin interaction, which, because of the involvement of *AXR1* in auxin signalling in many tissues, might be of general relevance for the whole plant.

### Root-specifically altered transcript levels under constitutive cytokinin deficiency

The transcript level of 79 genes was changed in a root-specific fashion: 35 genes were upregulated and 44 genes were downregulated (Additional file 3: Table S2). The strongest upregulation in roots showed a gene for an amino acid transporter family protein (AT5G65990) which was increased 18-fold (Table 2, Additional file 6: Figure S3a, b, Additional file 7: Figure S4a, b).

The transcript of the CLV3-related gene *CLE1* (AT1G73165) was upregulated 5.7-fold under cytokinin deficiency, however, the p-value (0.049) was slightly above our threshold (0.03). There is not much known about the actual function of *CLE1*. However, *CLE1* complemented the *clv3* mutant well and also requires CLV1 for signalling, and thus it may be considered a functional homologue [134]. The role of CLV3 and related CLE peptides in the shoot apical meristem has been investigated in great detail [135]. However, they are equally important in root meristem maintenance. Overexpression, as well as the exogenous application of several CLE peptides – including CLV3 – to roots, results in the consumption of root meristematic cells [136-140] and both CLV3 and CLE40, its functional orthologue in roots, promote differentiation in the distal root meristem. CLE40 forms a ligand-receptor pair with ACR4 to regulate *WOX5* expression, resulting in a similar regulatory loop for the balance between proliferation and differentiation as the CLV/WUS system in the shoot meristem [141]. It could be that the root-specific upregulation of *CLE1* under cytokinin deficiency is functionally relevant for the altered differentiation behaviour of cytokinin-deficient roots [24,26].

### Shoot-specifically altered transcript levels under constitutive cytokinin deficiency

363 genes were deregulated in a shoot-specific fashion in *35S:CKX1* transgenic seedlings, which is a more complex response compared to roots. 189 of the genes were upregulated and many showed very strong changes (~80-fold) of their expression level and a strong shift towards root-like expression (Additional file 3: Table S2) which has caused the shift of the cytokinin-deficient root sample towards a more root-like position in the PCA analysis discussed above. Interestingly, the three most strongly downregulated genes and two more genes with strongly reduced transcript levels encode AP2/ERF transcription factors (Additional file 3: Table S2, Additional file 6: Figure S3c, Additional file 7: Figure S4c, d), four of which are identical to those downregulated by cytokinin treatment (AT5G51190, AT5G61600, AT2G44840, and AT5G47220) mentioned above.

Several components of the light signalling network, in particular linked to phytochrome A, were found to be deregulated in cytokinin-deficient shoots. *SPA1* and *COP1* belong to the upregulated genes, while the *phyA* transcript itself is downregulated to one third of its level in wild-type plants (Additional file 3: Tables S2b and e). PhyA is involved in two light responses: the very-low-fluence response (VLFR) and the far-red-light-dependent high-irradiance response (HIR), the former promoting seed germination with very low quantities of red or far-red light and the latter promoting photomorphogenesis [142]. SPA1 (an atypical bHLH transcription factor) and COP1 (an E3 ubiquitin ligase) are negative regulators of partial aspects of both VLFR and HIR. COP1 and SPA1 are able to form a complex which targets PhyA for proteolytic degradation, thereby promoting photomorphogenesis [143]. The upregulation of the *COP1* and *SPA1* transcripts by cytokinin may contribute to the rapid degradation of PhyA in its P_*fr*_ form and the transcriptional downregulation of *PhyA* by cytokinin is likely to attenuate PhyA accumulation. Together, the gene regulation is consistent with altered light responses of cytokinin-deficient plants [27] and the known links between cytokinin and light responses [1,2].

Finally, the transcript of the *DIM*/*DWF1* gene encoding an enzyme involved in brassinosteroid biosynthesis [144] was downregulated to one third of its wild-type level in cytokinin-deficient shoots. *dim*/*dwf1* mutants are brassinosteroid-deficient and show the typical dwarfed phenotype. It would be interesting to study in what way an altered brassinosteroid level is causally involved in establishing the cytokinin deficiency syndrome of which reduced shoot growth is a hallmark [24]. In any case, together with the regulation of *CYP710A* and *CYP710B* genes mentioned above our results suggest an influence of cytokinin on brassinosteroid biosynthesis. Reciprocal influences of the two hormones on the expression of their metabolism genes have been observed before; however, the regulation of brassinosteroid metabolism genes by cytokinin was described as being ambiguous and not allowing a clear prediction whether this results in an enhanced or decreased brassinosteroid level [145].

## Conclusions

This study reports hitherto unknown differences of transcriptomic changes in root and shoot tissues in response to cytokinin treatment or cytokinin deficiency. One interesting result was the shift of long-term treated wild-type root samples to a more “shoot-like” profile and the opposite shift of cytokinin-deficient shoot samples towards “root-like” expression, both primarily affecting nuclear genes encoding plastid proteins. It would be interesting to analyze whether the whole transcriptome reacts in a rheostat-like fashion to a change in cytokinin status. There are some noteworthy examples of tissue-specific cytokinin responses, the most prominent probably being the cytokinin hydroxylase gene *CYP735A2*, which is strongly upregulated in root tissue but lacks a response in shoot tissue. This implies that strong negative regulators suppress the cytokinin response of *CYP735A2* in shoot tissue. Numerous novel cytokinin response genes were discovered which were missed previously because of unspecific sampling and it can be anticipated that studies with an even higher spatial resolution will continue to discover novel cytokinin-regulated genes in their functional context [146]. Several novel regulatory influences of cytokinin on metabolism and signalling genes of other hormonal regulators of plant development such as auxin and brassinosteroids were found. Similarly, novel connections between cytokinin and environmental cues operating through transcriptional changes were unravelled supporting the notion that the hormone is involved in regulating a vast array of plant functions [2]. Together these findings generate new hypotheses that need to be tested experimentally in order to fit them into the emerging picture of regulatory transcriptional networks [145,147].

## Methods

### Growth, treatment and harvest of *Arabidopsis thaliana*

*Arabidopsis thaliana* Col-0 seeds and seeds of ectopic overexpressors of the cytokinin oxidase/dehydrogenase gene *CKX1*[24] were surface-sterilized with chlorine gas [148]. Seedlings were grown before treatment for five days in liquid medium (half-strength MS medium, 1 g L^–1^ sucrose, 0.5 g L^–1^ MES, pH 5.7) under a 16 h light/8 h dark regime at 22°C. The whole seedlings were then treated with either 5 μM 6-benzyladenine (BA) for 15, 120 or 1080 min, or for 120 min in a solvent control, which were both added to the medium. To minimize circadian effects on gene expression, the treatment was timed in such a way that the harvest could be carried out for all samples between 6 h and 8 h after the onset of light. To achieve this, both the induction and the harvesting process for each sample covered the whole period of 2 h. At the end of the incubation period, roots and shoots were harvested separately by taking each seedling individually with a soft forceps at the hypocotyl, breaking off the roots of shock-frozen seedlings at the inner wall of a tube swimming in liquid nitrogen and collecting the remaining shoot in another tube. In this way, the breaking point was always very close to the root-hypocotyl junction and very pure root and shoot samples were obtained. The roots and shoots of at least 2,400 seedlings were pooled for one sample. Two samples were collected per condition.

RNA extraction, preparation of fluorescently labelled probes, hybridisation of microarrays and generation of raw data was performed as described previously [7]. Each sample was hybridized onto two microarrays, resulting in four microarrays per condition. The CATMA V2.3 microarray [149] of the Microarray Facility of the University Hospital Leuven (MAF, Leuven, Belgium, ArrayExpress accession no. A-MEXP-120) was used for this study. We followed the hybridization strategy used in the European Compendium of Arabidopsis Gene Expression project (http://www.cagecompendium.org), employing a Cy3-labeled mixture of oligonucleotides complementary to the secondary primers for probe amplification as a common reference sample and Cy5 labeling for the biological samples. This allowed for determination of the steady-state mRNA levels as opposed to the ratios usually obtained by two-color microarrays. The raw data are accessible at ArrayExpress (http://www.ebi.ac.uk/arrayexpress/) under the accession E-CAGE-111.

### Bioinformatical procedures

The raw data were normalized and pre-processed using the pre-processing pipeline developed with Bioconductor [150] in R [151] during the course of the CAGE project [152,153]. After pre-processing, linear models were fitted for each gene [154,155]. Linear modelling was performed using two factors: (1) organ with the levels “root” and “shoot”, and (2) cytokinin with the levels “CKX1”, “BA0”, “BA15”, “BA120”, and “BA1080”. From the individual *p*-values calculated in linear modelling, FDR-corrected *p*-values (“*q*-values”) were calculated [156]. Genes which were above background on less than four arrays were called “not expressed” and excluded from the analysis. Genes with less than ten spots above background exhibited a very high percentage of false positives, so only genes with at least ten spots above background were chosen for further analysis. The resulting dataset of (log) expressions, (log) ratios, *p*-values, *q*-values, and spots above background (AbB) was used to generate all further data derived.

Interaction plots were generated in R and reformatted with standard image editing software (CorelDraw).

Colour-coded expression maps (Figure 2a) were generated using Mapman [157,158]. Tables with log-ratios for the conditions compared were generated in R for this purpose and exported as tab-delimited text files in a Mapman-compatible format. Genes which were detected on less than two microarrays in the respective more highly expressed condition were flagged as not detected. The Mapman-generated images were exported and the colour-coded boxes were aligned using standard image editing software. Genes were categorized according to their expression patterns by an algorithm shown in Additional file 1: Figure S1a. Two sorting algorithms adapted to the datasets of the cytokinin-induced and the cytokinin-deficient plants, respectively, were implemented in R. This was done by splitting the dataset up into the subsets “root-specific”, “shoot-specific”, “differential”, “similar”, “uncategorized”, and “not regulated” using an algorithm outlined in Additional file 1: Figure S1.

Principal Component Analysis (PCA) based on the steady-state mRNA levels was carried out using the implementation in TIGR MEV 4 [159] with the standard settings using a restricted dataset of 911 genes with ≥ 10 spots above background and an FDR-corrected p-value of ≤ 0.0001. The graphic output was re-formatted using standard image editing software.

GO term enrichment was performed using the Gene Ontology website http://amigo.geneontology.org/cgi-bin/amigo/term_enrichment[160]. The graphic result was downloaded from the website and adapted to printing format. Normalization of the dataset for the organ effect was carried out using the following formula on the log-transformed expression values for the five shoot samples of each gene:

$$\begin{array}{l} norm_{shoot,condition}=raw_{shoot,sondition}+\left({r\bar{a}w}_{\mathrm{root}}-{r\bar{a}w}_{\mathrm{shoot}}\right)\\ norm_{root,condition}=raw_{root,condition} \end{array}$$

*norm*, normalized value; *raw*, native value; $r\bar{a}w$, average of the native values.

### Real-time quantitative PCR analysis

Gene expression profiles were verified by real-time quantitative PCR analysis using the following protocol. Each reaction contained 1× reaction buffer (10× stock, 160 mM (NH_4_)_2_SO_4_, 1 M Tris–HCl, pH 8.3, 0.1% Tween-20), 2 mM MgCl_2_, 100 μM each dNTP, 0.1× SYBR Green I (Fluka, St. Louis, MO, USA, 10,000× stock), 50 nM 5-carboxy-X-rhodamine triethylamine salt (ROX, Sigma, St. Louis, MO, USA), 50 – 300 nM of each primer, 10 mU/μL immolase (BioLine, Luckenwalde, Germany) in a total volume of 20 μL. Each sample-gene combination was run as triple replicates. Real-time PCR runs were performed in a 7500 Fast system (Applied Biosystems), controlled and evaluated by the 7500 Software V2.0.1 RC. In order to exclude primers binding to unspecific sequences, they were designed using GENOPLANTE™S.P.A.D.S (http://www.psb.ugent.be/SPADS/) [161] or NCBI Primer-BLAST (http://www.ncbi.nlm.nih.gov/tools/primer-blast/) [162]. The primer sequences are listed in Additional file 11: Table S7.

## Authors’ contributions

WGB carried out all the experimental and bioinformatical work, designed the research and bioinformatic approaches and contributed to the analysis of the data and writing the paper. Both read and approved the final manuscript.

## Supplementary Material

Additional file 1 **Figure S1.** Flow chart representing the algorithm to categorize genes detected in this microarray study. Schematic representation of the algorithm used for gene categorization. Only genes, which were detected on at least 25% of the microarrays were fed into the algorithm. Click here for file

Additional file 2 **Table S1.** Gene lists from cytokinin-treated samples. Lists of all genes detected on at least 25% of the microarrays (i.e. 8 arrays) containing expression data of wild-type samples sorted into the different categories as described in Methods and Additional file 1: Figure S1. The spreadsheet is divided into sections a-f containing genes which are root-specifically (a), shoot-specifically (b), differentially (c), or similarly (d) regulated by cytokinin. Section e contains cytokinin-regulated genes which are not unambiguously classifiable into one of the other categories. Section f contains genes not regulated by cytokinin. CATMA ID, unique identifier of the probe on the CATMA microarray; FDR p-value, p-value corrected for multiple testing; AGI, unique gene identifier assigned to *Arabidopsis thaliana* genes by TAIR. BA0, mock treatment; BA15, 15 min cytokinin treatment; BA120, 2 h cytokinin treatment; BA1080, 18 h cytokinin treatment. The sorting criteria were (a) the sum of all ratios in the root; (b) the sum of all ratios in the shoot; (c) the sum of the absolute value of the log2 of all ratios in root and shoot; (d) the sum of all ratios in root and shoot; (e) the same as for (c); (f) CATMA ID. Click here for file

Additional file 3 **Table S2.** Gene lists from cytokinin-deficient samples. Lists of all genes detected on at least 25% of the microarrays (i.e. 4 arrays) containing expression data of mock-treated wild-type samples (BA0) and samples of cytokinin-deficient plants (CKX1), sorted into the different categories as described in Methods and Additional file 1: Figure S1. The spreadsheet is divided into sections a-f containing genes which are root-specifically (a), shoot-specifically (b), differentially (c), or similarly (d) regulated. Section e contains cytokinin-regulated genes which are not unambiguously classifiable into one of the other categories. Section f contains genes not found regulated by cytokinin. CATMA ID, unique identifier of the probe on the CATMA microarray; FDR p-value, p-value corrected for multiple testing; AGI, unique gene identifier assigned to *Arabidopsis thaliana* genes by TAIR. The sorting criteria were (a) the ratio CKX1 vs. Col-0 root; (b) the ratio CKX1 vs. Col-0 shoot; (c) the sum of the absolute value of the log2 of both ratios; (d) the sum of both CKX1 vs. Col-0 ratios; (e) the same as for (c); (f) CATMA ID. Click here for file

Additional file 4 **Table S3.** Genes expressed in a more shoot-like fashion in cytokinin-treated roots. (a) List of genes, which are higher expressed in the shoot than in the root and upregulated by 18 h of cytokinin treatment in the root. (b) List of genes, which are lower expressed in the shoot than in the root and downregulated by 18 h of cytokinin treatment in the root. FDR-corrected p-values ≤ 0.03 marked in blue indicate whether a gene is significantly regulated by cytokinin (cytokinin effect), significantly differently expressed roots and shoots (organ effect), or whether its regulation by cytokinin differs significantly between roots and shoots (organ effect). The threshold set for differential expression is 2.5-fold. Click here for file

Additional file 5 **Figure S2.** GO term enrichment for genes indicative of a developmental shift. (a) GO term enrichment of genes which are more shoot-like expressed in cytokinin-induced roots. The figure shows that the only overrepresented class of genes encodes proteins located in the chloroplast. The dataset of Additional file 4: Table 3a was used to generate this figure. Darker boxes indicate a higher significance of overrepresentation of the GO term indicated. (b) GO term enrichment of genes which are more root-like expressed in cytokinin-deficient shoots. The figure shows that the only overrepresented class of genes encodes proteins located in the chloroplast. The dataset of Additional file 4: Table 3b was used to generate this figure using the AmiGO tool (see Methods). Click here for file

Additional file 6 **Figure S3.** Expression of selected organ-specific cytokinin-regulated genes mentioned in the text as revealed by microarray analysis and real-time quantitative RT-PCR. (a) Differentially expressed genes after cytokinin induction. (b) Expression ratios of untreated shoots and roots of the genes shown in (a). (c) Differentially expressed genes under cytokinin deficiency. (d) Expression ratios in wild-type shoots and roots of the genes shown in (c). Cytokinin regulation of selected genes with different organ specificity of the response was tested by microarrays (indicated by diamonds) and qRT-PCR (indicated by bars) using independent biological material. BD, below detection limit of microarray; bd, below detection limit of qRT-PCR. Click here for file

Additional file 7 **Figure S4.** Expression levels of selected organ-specific cytokinin-regulated genes mentioned in the text as revealed by microarray analysis and real-time quantitative RT-PCR. (a, b) Expression levels of differentially expressed genes after cytokinin treatment. (c, d) Expression levels of differentially expressed genes under cytokinin-deficiency. The expression levels of selected genes with different organ specificity of the response were tested under the conditions indicated in the legend by microarray analysis (a, c) and qRT-PCR (b, d) using independent biological material. Open diamonds indicate that less than two of the four spots on the microarray were significantly above background. nd, not detected. Click here for file

Additional file 8 **Table S4.** Genes expressed in a more root-like fashion in cytokinin-deficient shoots. (a) List of genes, which are higher expressed in the root than in the shoot and upregulated by chronic cytokinin deficiency in the shoot. (b) List of genes, which are lower expressed in the root than in the shoot and downregulated by chronic cytokinin deficiency in the shoot. FDR-corrected p-values ≤ 0.03 marked in blue indicate whether a gene is significantly regulated by cytokinin (cytokinin effect), significantly differently expressed roots and shoots (organ effect), or whether its regulation by cytokinin differs significantly between roots and shoots (organ effect). The threshold set for differential expression is 2.5-fold. Click here for file

Additional file 9 **Table S5.** Root- and shoot-specific changes of transcript abundance of known cytokinin-regulated genes of *Arabidopsis thaliana*. A selection of previously published cytokinin-responsive genes from various publications were analyzed for their transcriptional regulation in response to cytokinin induction and cytokinin deficiency in root and shoot samples. Expression ratios are colour-coded as described for Table 1. Significance codes indicate p-values calculated as described in Methods: 0.1 > · > 0.05 > * 0.01 > ** 0.001 > ***. Genes that were published in at least two previous publications are listed here. In addition, selected examples of known regulated genes that were only published once were added. A short list of genes can be found in Table 2. The publications are coded by letters: A, Rashotte et al., 2003 [15]; B, Brenner et al., 2005 [9]; C, Kiba et al., 2005 [13]; D, Rashotte et al., 2006 [16]; E, Taniguchi et al., 2007 [17]; F, Argyros et al., 2008 [6]. AGI, unique gene identifier assigned to *Arabidopsis thaliana* genes by TAIR. (PDF 336 kb) Click here for file

Additional file 10 **Table S6.** Cytokinin-responsive genes encoding ribosomal proteins. The table shows all cytokinin-regulated genes encoding ribosomal protein according to the GO localization database. Columns and colour-codes are the same as for Table 2. The additional column localization shows whether the protein is part of a plastid or cytosolic ribosome, according to the GO database (http://www.arabidopsis.org/tools/bulk/go/index.jsp). Click here for file

Additional file 11 **Table S7.** Primer sequences used for qRT-PCR. The primer sequences were selected using GENOPLANTE™S.P.A.D.S. (http://www.psb.ugent.be/SPADS/, grey background) or NCBI Primer-BLAST (http://www.ncbi.nlm.nih.gov/tools/primer-blast/). Click here for file
