# Supplementary material for: Transcript profiling of cytokinin action in Arabidopsis roots and shoots discovers largely similar but also organ-specific responses
Source: BMC Plant Biol. 2012 Jul 23;12:112. doi: 10.1186/1471-2229-12-112 (PMC3519560; doi:10.1186/1471-2229-12-112)
Supplement: Additional file 1 — Figure S1. Flow chart representing the algorithm to categorize genes detected in this microarray study. Schematic representation of the algorithm used for gene categorization. Only genes, which were detected on at least 25% of the microarrays were fed into the algorithm. [file 1471-2229-12-112-S1.pdf]

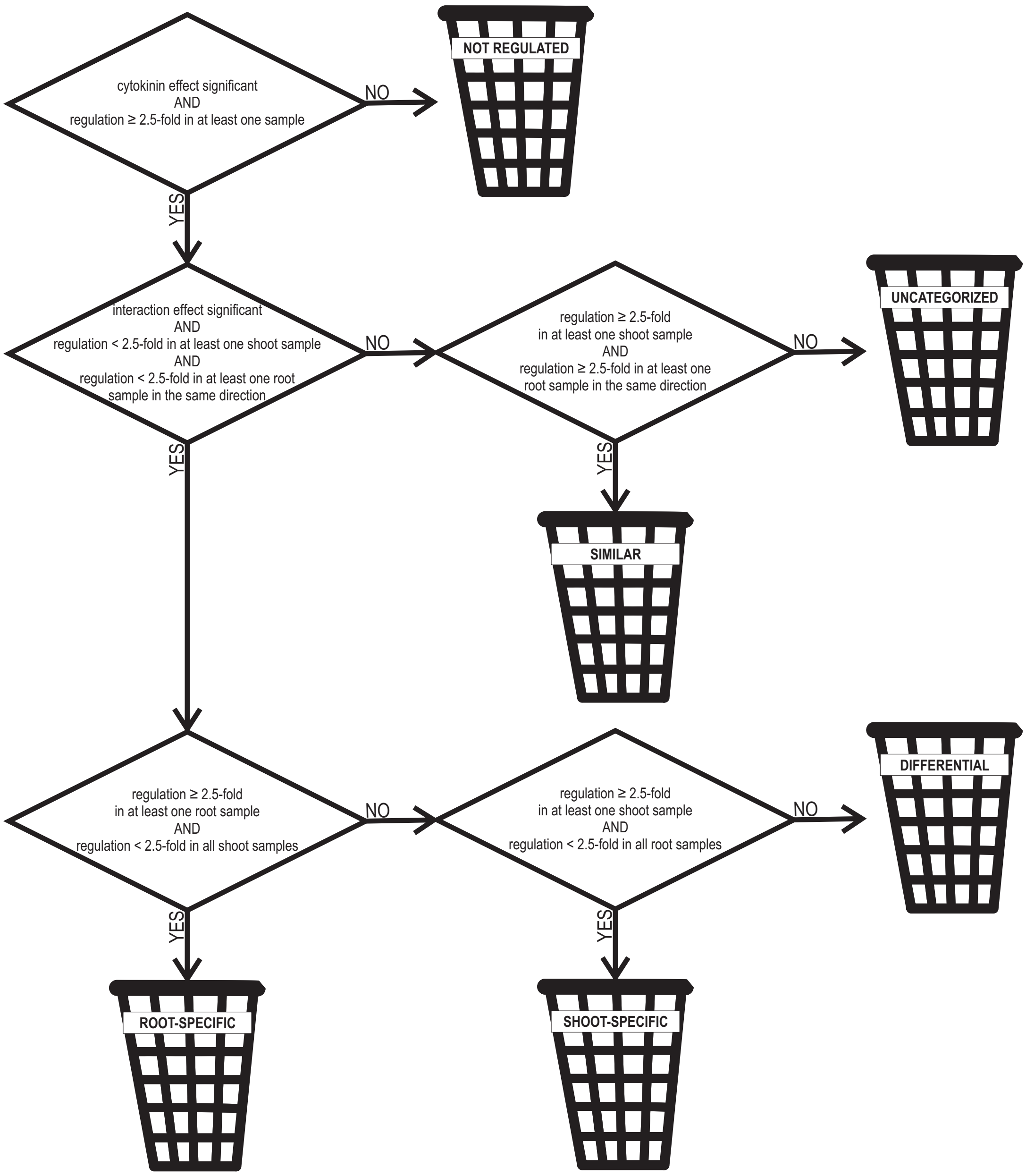

Supplemental Figure 1. Flow chart representing the algorithm to categorize genes detected in this microarray study. Schematic representation of the algorithm used for gene categorization. Only genes, which were detected on at least 25% of the microarrays were fed into the algorithm.
