# Supplementary material for: Transcript profiling of cytokinin action in Arabidopsis roots and shoots discovers largely similar but also organ-specific responses
Source: BMC Plant Biol. 2012 Jul 23;12:112. doi: 10.1186/1471-2229-12-112 (PMC3519560; doi:10.1186/1471-2229-12-112)
Supplement: Additional file 5 — Figure S2. GO term enrichment for genes indicative of a developmental shift. (a) GO term enrichment of genes which are more shoot-like expressed in cytokinin-induced roots. The figure shows that the only overrepresented class of genes encodes proteins located in the chloroplast. The dataset of Additional file 4: Table 3a was used to generate this figure. Darker boxes indicate a higher significance of overrepresentation of the GO term indicated. (b) GO term enrichment of genes which are more root-like expressed in cytokinin-deficient shoots. The figure shows that the only overrepresented class of genes encodes proteins located in the chloroplast. The dataset of Additional file 4: Table 3b was used to generate this figure using the AmiGO tool (see Methods). [file 1471-2229-12-112-S5.pdf]

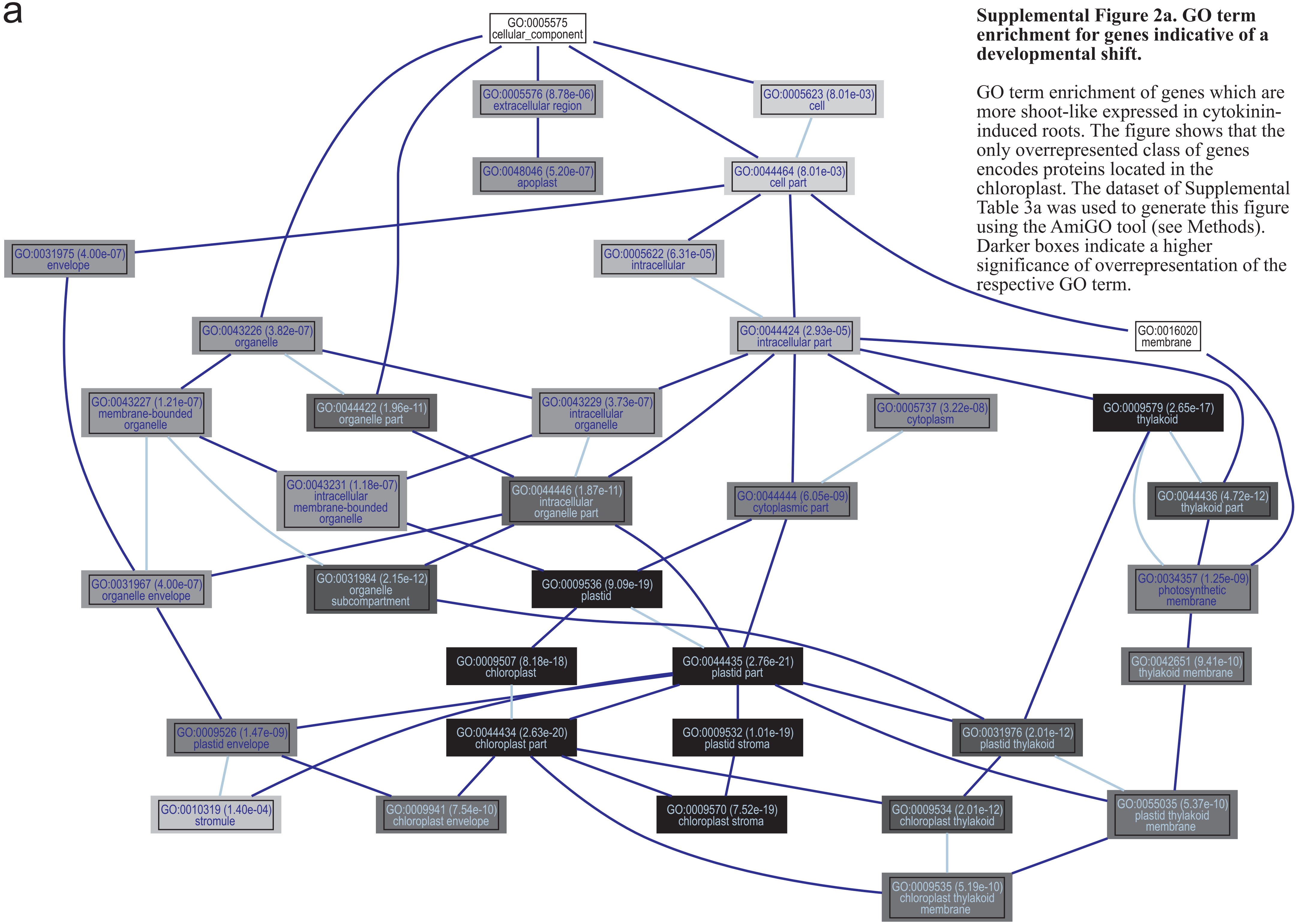


**Supplemental Figure 2a. GO term enrichment for genes indicative of a developmental shift.**

GO term enrichment of genes which are more shoot-like expressed in cytokinin-induced roots. The figure shows that the only overrepresented class of genes encodes proteins located in the chloroplast. The dataset of Supplemental Table 3a was used to generate this figure using the AmiGO tool (see Methods). Darker boxes indicate a higher significance of overrepresentation of the respective GO term.

b

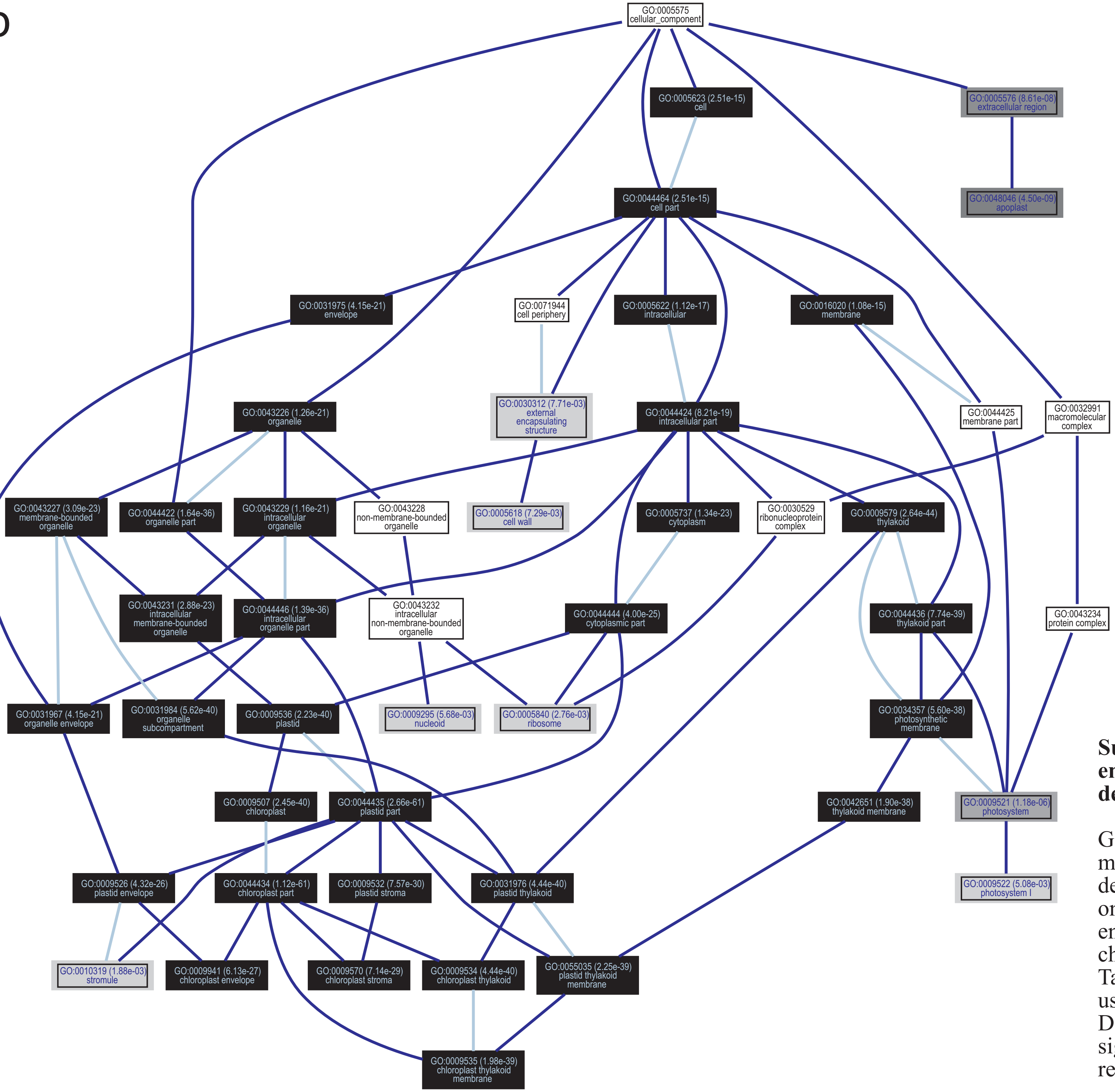


**Supplemental Figure 2b. GO term enrichment for genes indicative of a developmental shift.**

GO term enrichment of genes which are more root-like expressed in cytokinin-deficient shoots. The figure shows that the only overrepresented class of genes encodes proteins located in the chloroplast. The dataset of Supplemental Table 4b was used to generate this figure using the AmiGO tool (see Methods). Darker boxes indicate a higher significance of overrepresentation of the respective GO term.
