# Supplementary material for: Transcript profiling of cytokinin action in Arabidopsis roots and shoots discovers largely similar but also organ-specific responses
Source: BMC Plant Biol. 2012 Jul 23;12:112. doi: 10.1186/1471-2229-12-112 (PMC3519560; doi:10.1186/1471-2229-12-112)
Supplement: Additional file 6 — Figure S3. Expression of selected organ-specific cytokinin-regulated genes mentioned in the text as revealed by microarray analysis and real-time quantitative RT-PCR. (a) Differentially expressed genes after cytokinin induction. (b) Expression ratios of untreated shoots and roots of the genes shown in (a). (c) Differentially expressed genes under cytokinin deficiency. (d) Expression ratios in wild-type shoots and roots of the genes shown in (c). Cytokinin regulation of selected genes with different organ specificity of the response was tested by microarrays (indicated by diamonds) and qRT-PCR (indicated by bars) using independent biological material. BD, below detection limit of microarray; bd, below detection limit of qRT-PCR. [file 1471-2229-12-112-S6.pdf]

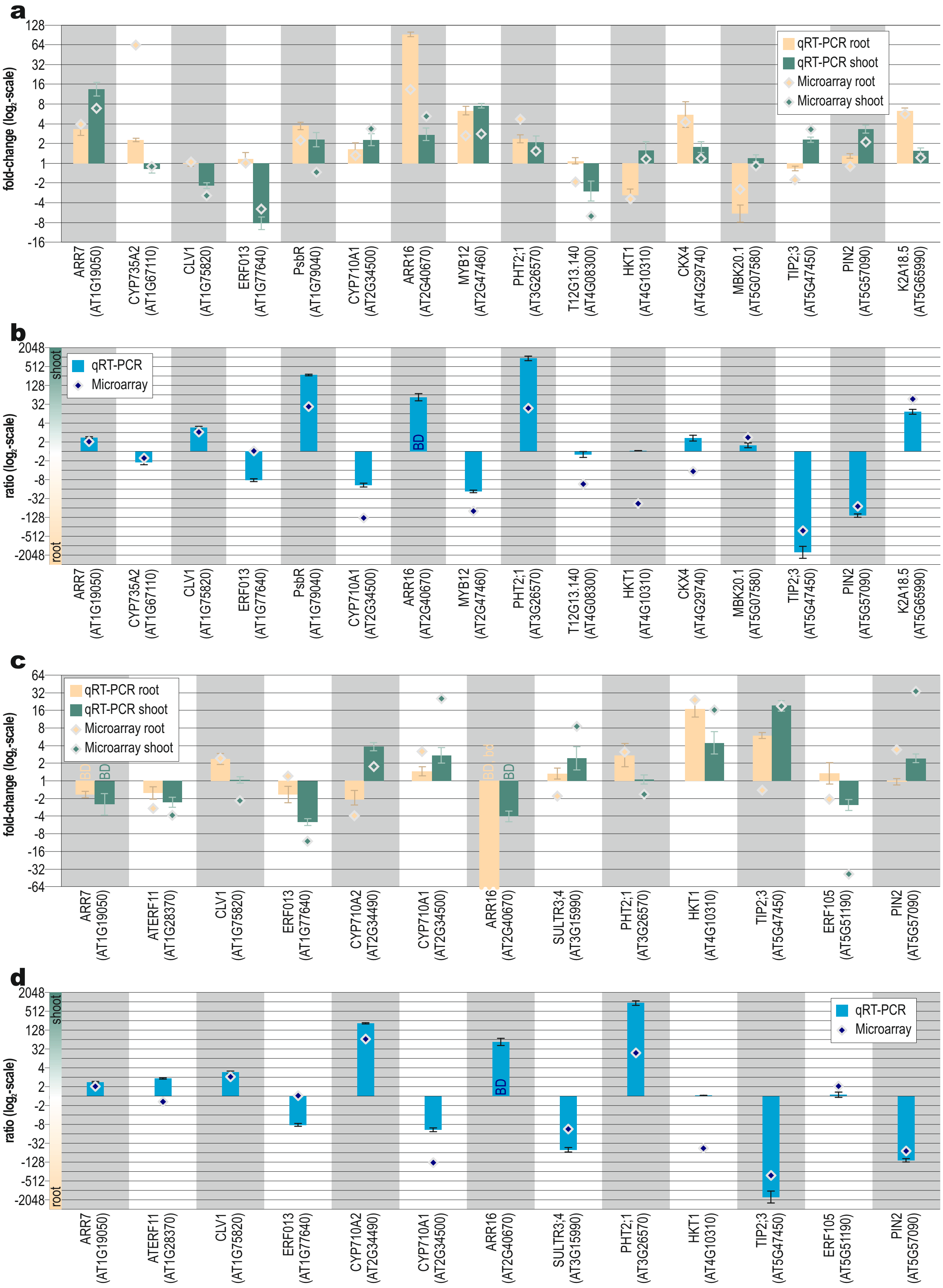

Supplemental Figure 3. Expression of selected organ-specific cytokinin-regulated genes mentioned in the text as revealed by microarray analysis and real-time quantitative RT-PCR. (a) Differentially expressed genes after cytokinin induction. (b) Expression ratios of untreated shoots and roots of the genes shown in (a). (c) Differentially expressed genes under cytokinin deficiency. (d) Expression ratios in wild-type shoots and roots of the genes shown in (c). Cytokinin regulation of selected genes with different organ specificity of the response was tested by microarrays (indicated by diamonds) and qRT-PCR (indicated by bars) using independent biological material. BD, below detection limit of microarray; bd, below detection limit of qRT-PCR.
