# Supplementary material for: Transcript profiling of cytokinin action in Arabidopsis roots and shoots discovers largely similar but also organ-specific responses
Source: BMC Plant Biol. 2012 Jul 23;12:112. doi: 10.1186/1471-2229-12-112 (PMC3519560; doi:10.1186/1471-2229-12-112)
Supplement: Additional file 7 — Figure S4. Expression levels of selected organ-specific cytokinin-regulated genes mentioned in the text as revealed by microarray analysis and real-time quantitative RT-PCR. (a, b) Expression levels of differentially expressed genes after cytokinin treatment. (c, d) Expression levels of differentially expressed genes under cytokinin-deficiency. The expression levels of selected genes with different organ specificity of the response were tested under the conditions indicated in the legend by microarray analysis (a, c) and qRT-PCR (b, d) using independent biological material. Open diamonds indicate that less than two of the four spots on the microarray were significantly above background. nd, not detected. [file 1471-2229-12-112-S7.pdf]

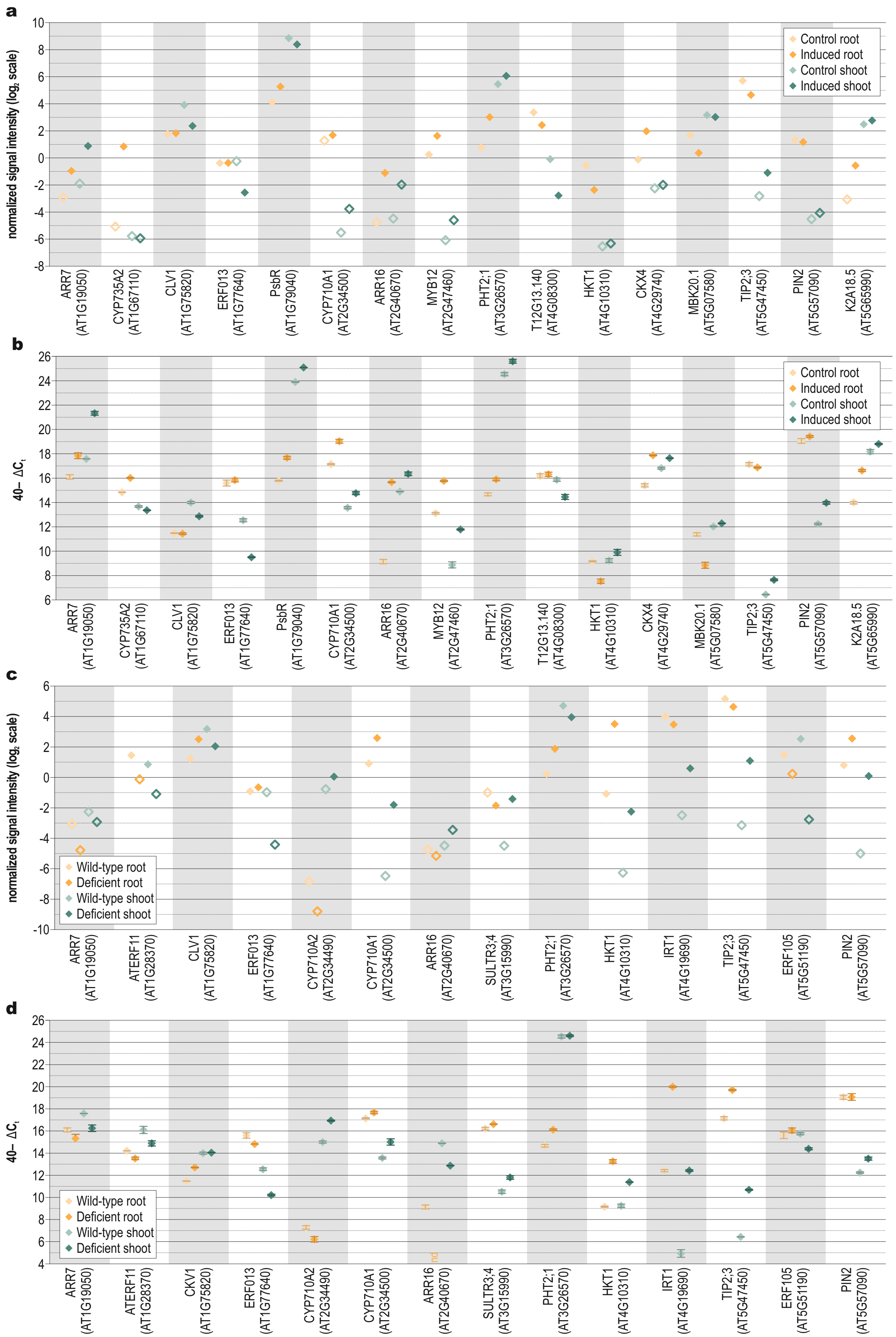

Supplemental Figure 4. Expression levels of selected organ-specific cytokinin-regulated genes mentioned in the text as revealed by microarray analysis and real-time quantitative RT-PCR. (a, b) Expression levels of differentially expressed genes after cytokinin treatment. (c, d) Expression levels of differentially expressed genes under cytokinin-deficiency. The expression levels of selected genes with different organ specificity of the response were tested under the conditions indicated in the legend by microarray analysis (a, c) and qRT-PCR (b, d) using independent biological material. Open diamonds indicate that less than two of the four spots on the microarray were significantly above background. nd, not detected.
