# Supplementary material for: Transcript profiling of cytokinin action in Arabidopsis roots and shoots discovers largely similar but also organ-specific responses
Source: BMC Plant Biol. 2012 Jul 23;12:112. doi: 10.1186/1471-2229-12-112 (PMC3519560; doi:10.1186/1471-2229-12-112)
Supplement: Additional file 9 — Table S5. Root- and shoot-specific changes of transcript abundance of known cytokinin-regulated genes of Arabidopsis thaliana. A selection of previously published cytokinin-responsive genes from various publications were analyzed for their transcriptional regulation in response to cytokinin induction and cytokinin deficiency in root and shoot samples. Expression ratios are colour-coded as described for Table 1. Significance codes indicate p-values calculated as described in Methods: 0.1 > · > 0.05 > * 0.01 > ** 0.001 > ***. Genes that were published in at least two previous publications are listed here. In addition, selected examples of known regulated genes that were only published once were added. A short list of genes can be found in Table 2. The publications are coded by letters: A, Rashotte et al., 2003 [15]; B, Brenner et al., 2005 [9]; C, Kiba et al., 2005 [13]; D, Rashotte et al., 2006 [16]; E, Taniguchi et al., 2007 [17]; F, Argyros et al., 2008 [6]. AGI, unique gene identifier assigned to Arabidopsis thaliana genes by TAIR. (PDF 336 kb) [file 1471-2229-12-112-S9.pdf]

Supplemental Table 4. Root- and shoot-specific changes of transcript abundance of known cytokinin-regulated genes of *Arabidopsis thaliana*. A selection of previously published cytokinin-responsive genes from various publications were analyzed for their transcriptional regulation in response to cytokinin induction and cytokinin deficiency in root and shoot samples. Expression ratios are colour-coded as described for Table 1. Significance codes indicate p-values calculated as described in Materials and Methods: 0.1 > • > 0.05 > * 0.01 > ** 0.001 > ***. Genes that were published in at least two previous publications are listed here. In addition, selected examples of known regulated genes that were only published once were added. A short list of genes can be found in Table 2. The publications are coded by letters: A, Rashotte et al., 2003; B, Brenner et al., 2005; C, Kiba et al., 2005; D, Rashotte et al., 2006; E, Taniguchi et al., 2007; F, Argyros et al., 2008. AGI, unique gene identifier assigned to *Arabidopsis thaliana* genes by TAIR.

Category	AGI	Description	Ratio								Significance codes		Previously published in
			Root				Shoot						
			CKX1 vs. BA0	BA15 vs. BA0	BA120 vs. BA0	BA1080 vs. BA0	CKX1 vs. BA0	BA15 vs. BA0	BA120 vs. BA0	BA1080 vs. BA0	Cytokinin effect	Interaction effect	
root-specific	AT2G40230	transferase family	1.07	0.81	2.72	2.99	2.32	0.93	1.18	2.13	**	.	A B C E
	AT1G67110	cytochrome P450, putative	0.61	4.20	60.65	29.72	2.09	1.05	0.90	2.06	***	***	A C E F
	AT2G01830	histidine kinase (CRE1/AHK4)	0.64	1.39	4.14	0.77	1.60	1.38	0.83	1.92		*	A C F
	AT4G16990	similar to disease resistance protein (TIR-NBS-LRR class)	0.13	0.60	0.47	2.63	0.87	0.57	1.02	0.88	*	*	A F
	AT4G19170	9-cis-epoxycarotenoid dioxygenase, putative	0.93	0.30	0.18	1.08	1.55	0.68	0.59	0.64	**		A F
	AT1G17190	Glutathione S-transferase	1.77	2.85	7.63	1.07	1.75	1.20	0.77	0.56	*	*	C F
	AT5G47980	Transferase family	0.35	1.61	6.18	14.36	0.82	0.47	0.56	2.00	**	*	C F
	AT2G44940	ERF/AP2 subfamily DREB / A-4 member	0.74	0.65	2.86	2.59	1.07	0.67	1.23	1.73	**		A
	AT1G78860	curculin-like (mannose-binding) lectin family	1.07	1.32	0.40	0.76	0.60	1.09	0.91	0.42	***	**	A
	AT2G42840	protodermal factor 1 (PDF1)	8.78	1.90	0.70	0.61	1.12	1.30	0.82	1.14	***	**	A
	AT3G47380	invertase/pectin methylesterase inhibitor family	0.64	1.20	2.83	11.71	1.18	0.58	0.52	1.80	***	***	A
	AT3G60280	uclacyanin 3 (UCC3)	1.18	0.58	2.23	5.32	1.43	0.47	0.88	1.21	***	*	A
	AT4G04840	methionine sulfoxide reductase domain-containing protein	3.87	2.05	1.59	8.46	0.42	1.80	1.16	1.84	***	**	A
	AT4G37160	multi-copper oxidase type I family	3.29	2.42	0.81	0.43	1.35	1.12	1.12	1.19	***	**	A
	AT1G72140	proton-dependent oligopeptide transport (POT) family	0.13	1.38	3.37	10.74	0.71	2.35	2.12	0.99	***	***	C
	AT3G03920	Gar1 RNA-binding region family	1.14	1.06	0.79	2.86	0.45	1.08	1.29	1.85	***	.	C
	AT3G29030	Expansin (EXP5)	0.04	0.96	0.62	1.21	0.66	0.76	0.85	2.25	***	*	C
	AT4G20440	Small nuclear ribonucleoprotein-associated protein B	4.44	2.02	1.41	2.26	1.50	1.26	1.79	1.45	***	*	C
	AT2G04795	expressed protein	0.66	0.68	0.24	1.13	0.49	0.46	0.59	1.19	***	*	F
	AT3G59400	expressed protein	4.28	1.23	0.63	3.83	0.70	0.87	0.93	0.81	***	***	F
	AT5G06530	ABC transporter family	19.98	0.82	0.91	0.90	2.46	1.24	1.91	0.67	***	*	F
	AT5G24030	C4-dicarboxylate transporter/malic acid transport family	4.19	1.54	2.2	0.35	2.29	1.60	1.09	1.72	***	**	F
shoot-specific	AT2G28950	expansin, putative (EXP6)	1.47	0.87	0.62	1.05	0.30	0.64	0.56	0.63		*	A C
	AT3G48750	A-type cyclin-dependent kinase	0.95	1.00	1.40	0.65	4.01	8.78	7.92	1.84	.		A E
	AT3G62930	Glutaredoxin family	1.97	2.10	2.06	2.10	0.36	0.88	1.58	2.03	*	.	C E
	AT3G21670	Nitrate transporter (NTP3)	0.84	0.70	0.72	1.36	2.00	2.66	2.65	4.47	*		C F
	AT1G10820	expressed protein	1.20	1.06	1.27	0.76	2.51	0.81	1.10	0.91	***	**	A
	AT1G55670	photosystem I reaction center subunit V, putative (PSAG)	1.50	1.29	0.70	2.28	0.05	0.71	0.39	0.50	***	***	A
	AT2G30570	photosystem II reaction center W (PsbW) related	1.49	0.62	0.46	2.41	0.15	0.41	0.30	0.70	***	***	A
	AT2G43540	expressed protein	1.55	0.63	0.54	1.82	0.23	0.31	0.39	0.49	***	***	A
	AT2G46690	auxin-responsive family	1.04	0.79	0.54	0.48	6.82	0.92	1.57	1.43	***	**	A
	AT2G47160	anion exchange family	0.75	0.61	0.54	0.68	1.39	0.36	0.36	0.74	***	.	A
	AT2G20940	Expressed protein	1.09	0.65	0.47	2.29	0.25	0.79	0.99	1.20	***	**	C
	AT3G44750	Histone deacetylase (HD2A)	2.32	1.85	0.95	1.04	6.39	2.30	2.19	3.61	***	.	C
	AT3G61100	Expressed protein	0.79	0.86	0.66	1.75	0.32	1.08	1.46	1.86	***	**	C
	AT4G34950	Nodulin family	1.26	0.75	2.36	1.28	0.19	0.38	0.42	0.94	***	***	C
	AT5G15210	Zinc finger homeobox family protein/ZF-HD homeobox family	0.71	0.60	1.03	2.10	0.36	0.87	0.96	1.03	***	*	C
	AT5G62440	Expressed protein	1.69	1.55	1.19	0.85	3.24	1.36	4.07	2.05	**	.	C
	AT5g53290	ERF/AP2 subfamily B-5 member (CRF3)	1.00	2.28	1.71	0.79	3.03	5.47	7.94	6.26	***	***	D
	AT1G70230	expressed protein	1.89	1.11	1.37	0.54	3.37	2.72	3.89	0.70	***		F
	AT2G30520	signal transducer of phototropic response (RPT2)	2.14	1.12	0.86	1.94	1.30	0.96	0.84	2.5	***		F
in both organs	AT1G69530	expansin (At-EXP1)	0.89	1.52	2.18	13.23	0.93	1.00	1.27	4.16	***		A B C E
	AT4G29740	cytokinin oxidase family (CKX4)	0.09	0.43	4.30	2.88	0.83	1.20	1.90	4.44	***	**	A C E F
	AT1G19050	A-type response regulator (ARR7)	0.31	4.53	14.48	0.97	0.63	2.57	9.23	2.07	***		A B F

Category	AGI	Description	Ratio: Root CKX1 vs. BA0	Root BA15 vs. BA0	Root BA120 vs. BA0	Root BA1080 vs. BA0	Shoot CKX1 vs. BA0	Shoot BA15 vs. BA0	Shoot BA120 vs. BA0	Shoot BA1080 vs. BA0	Significance codes: Cytokinin effect	Significance codes: Interaction effect	Previously published in
	AT2G40670	A-type response regulator (ARR16)	0.75	2.73	12.36	1.58	2.04	0.82	4.93	5.68	*		A B F
	AT1G10470	A-type response regulator (ARR4)	0.21	0.64	2.53	2.10	0.27	0.93	1.20	1.61	***		A B
	AT2G33850	expressed protein	9.59	1.31	0.53	3.98	3.33	2.41	1.77	1.53	**		A B
	AT1G67740	photosystem II core complex protein (PSBY)	2.72	1.30	0.13	5.63	1.35	1.35	0.58	0.4	***	***	A C
	AT2G36870	xyloglucan:xyloglucosyl transferase, putative	1.73	0.43	1.86	3.69	0.23	0.61	0.82	1.24	***	***	A C
	AT2G20520	fasciclin-like arabinogalactan-protein (FLA6)	1.65	0.54	1.97	6.21	4.24	1.18	2.64	1.00	.	.	A E
	AT4G10120	sucrose-phosphate synthase, putative	4.24	1.20	0.41	5.24	2.76	1.38	1.87	0.71	.	.	A F
	AT4G30270	xyloglucan endo-1,4-beta-D-glucanase (SEN4/MERI5B)	1.38	0.30	0.13	0.50	0.06	0.32	0.26	0.44	***	***	A F
	AT4G27410	no apical meristem (NAM) family (RD26)	0.78	0.60	1.68	2.62	0.44	0.70	0.85	0.82	*		B F
	AT1G13420	Sulfotransferase family protein	0.48	0.68	15.48	1.41	5.43	1.82	0.59	1.00		**	C F
	AT3G50300	Transferase family protein	1.92	1.91	5.25	3.00	2.38	0.12	0.28	1.45		.	C F
	AT3G57010	Strictosidine synthase family	1.24	1.41	7.99	4.19	9.13	2.47	4.27	7.07	***	*	C F
	AT5G42590	Cytochrome P450 71A16, putative (CYP71A16)	0.76	0.77	11.73	1.74	1.14	0.36	0.38	0.54	**	***	C F
	AT5G51440	Mitochondrial small heat shock protein (HSP23.5-M)	15.18	3.34	3.32	5.58	0.88	1.42	14.16	2.29	*	*	C F
	AT1G43160	ERF/AP2 subfamily B-4 member (RAP2.6)	0.71	1.79	2.81	0.59	2.56	2.96	1.07	3.45			A
	AT2G40340	ERF/AP2 subfamily DREB / A-2 member	0.38	1.57	1.14	1.54	0.40	0.86	0.97	0.96	***		A
	AT4G34410	ERF/AP2 subfamily B-3 member (RRTF1)	0.18	0.33	0.19	0.06	0.46	0.25	1.03	0.48	*		A
	AT5G47220	ERF/AP2 subfamily B-3 member (ATERF-2)	0.69	0.60	0.44	0.11	0.10	0.48	0.18	0.07	***	**	A
	AT5G13330	ERF/AP2 subfamily B-4 member (RAP2.6L)	0.87	0.92	0.87	1.24	0.68	0.86	0.53	2.38	*		B
	AT1G09770	myb family transcription factor	0.37	1.23	1.37	0.51	1.78	1.95	4.54	1.80	***	*	A
	AT1G20340	plastocyanin	2.67	0.70	0.47	3.21	0.14	0.33	0.28	0.65	***	***	A
	AT1G61520	chlorophyll A-B binding protein (LHCA3.1)	2.06	1.29	0.75	3.72	0.24	0.43	0.41	0.77	***	***	A
	AT1G61590	protein kinase, putative	0.80	0.52	0.10	0.58	1.71	0.16	0.38	2.47	***	*	A
	AT1G71030	myb family transcription factor, putative	0.59	0.86	0.11	1.23	0.29	0.37	0.20	0.55	***	*	A
	AT1G72830	CCAAT-binding transcription factor (CBF-B/NF-YA) family	0.37	0.55	0.83	0.17	10.06	0.44	1.73	1.28	***	***	A
	AT1G75820	CLAVATA1 receptor kinase (CLV1)	2.63	1.05	0.79	0.54	0.42	0.34	0.32	0.09	***	***	A
	AT2G15890	expressed protein	0.36	0.69	0.19	0.60	0.09	0.25	0.20	0.22	***	*	A
	AT2G17880	DNAJ heat shock protein	0.44	0.72	0.13	0.75	0.09	0.49	0.09	0.11	***	*	A
	AT2G28160	putative transcription factor that regulates iron uptake responses	0.68	0.64	1.06	0.10	3.79	0.47	0.59	0.41	***	*	A
	AT2G40460	proton-dependent oligopeptide transport (POT) family	5.13	1.36	1.68	0.41	7.26	1.46	1.58	0.52	***		A
	AT3G12500	basic endochitinase	7.75	1.02	0.85	1.87	3.86	0.38	0.53	0.22	***		A
	AT3G21055	photosystem II 5 kD protein, putative	0.58	0.44	0.16	1.05	0.11	0.42	0.28	0.39	***	*	A
	AT4G08290	nodulin MtN21 family	0.41	0.8	0.59	0.31	2.32	1.76	1.08	0.23	***	***	A
	AT4G10340	chlorophyll A-B binding protein CP26 (LHCB5)	1.18	0.52	0.36	1.91	0.22	0.40	0.44	0.93	***	**	A
	AT4G13770	cytochrome P450 family	2.00	1.88	1.08	0.23	7.15	10.52	4.17	2.70	***	.	A
	AT4G14130	xyloglucan:xyloglucosyl transferase, putative (XTR7)	1.69	1.18	0.33	0.44	0.49	0.56	0.33	0.28	***	.	A
	AT4G17340	major intrinsic family protein / MIP family	0.96	0.67	0.66	0.07	4.75	2.61	0.60	0.22	***	.	A
	AT4G28270	zinc finger (C3HC4-type RING finger) family	0.96	0.59	0.16	0.85	0.49	0.37	0.32	0.33	***	**	A
	AT4G29020	glycine-rich protein	5.03	0.43	0.29	2.58	0.42	0.41	0.33	0.56	***	**	A
	AT4G35160	O-methyltransferase family 2	0.09	1.73	5.22	3.10	0.29	0.95	1.55	0.69	***	*	A
	AT5G13930	chalcone synthase / naringenin-chalcone synthase	3.96	1.32	0.51	2.71	4.06	1.95	1.29	1.23	***	**	A
	AT5G19890	peroxidase, putative	2.28	1.44	0.64	0.18	3.45	0.59	0.53	0.33	***		A
	AT5G23380	expressed protein	1.32	1.15	0.99	4.43	0.90	0.79	0.29	0.74	***	**	A
	AT5G57090	auxin transport protein (EIR1)	3.66	2.12	0.89	0.59	35.26	1.36	1.15	0.86	***	*	A

Category	AGI	Description	Ratio								Significance codes		Previously published in
			Root				Shoot						
			CKX1 vs. BA0	BA15 vs. BA0	BA120 vs. BA0	BA1080 vs. BA0	CKX1 vs. BA0	BA15 vs. BA0	BA120 vs. BA0	BA1080 vs. BA0	Cytokinin effect	Interaction effect	
	AT1G03850	glutaredoxin family protein	0.25	0.99	0.45	1.02	0.38	1.24	0.66	0.84	***		B
	AT1G21830	expressed protein	0.38	0.87	0.75	1.70	0.29	0.64	0.67	0.93	***		B
	AT1G49500	expressed protein	0.36	0.83	0.72	1.82	0.06	0.54	0.41	0.43	***	*	B
	AT1G56150	auxin-responsive family protein	0.74	0.49	0.33	1.32	0.14	0.37	0.19	0.38	***	.	B
	AT2G02310	F-box family	0.57	1.38	1.58	1.50	0.32	1.22	0.72	1.00	***		B
	AT4G34750	small auxin up RNA (SAUR_E)	0.79	1.02	1.05	1.99	0.34	0.62	0.69	0.94	***		B
	AT5G20230	plastocyanin-like domain-containing protein	0.22	0.49	1.09	0.35	0.06	0.41	0.25	0.89	***	*	B
	AT5G62920	A-type response regulator (ARR6)	0.12	1.62	2.35	1.73	0.26	1.28	1.72	0.96	***	.	B
	AT1G20190	Expansin, putative (EXP11)	0.36	0.52	0.94	0.40	0.26	0.85	0.47	0.26	***	**	C
	AT1G58170	Disease resistance-responsive protein-related	2.85	1.81	8.58	7.94	1.24	2.28	5.91	9.68	***		C
	AT2G22770	bHLH family transcription factor (AtbHLH20)	0.09	1.15	2.82	2.98	0.40	1.02	0.83	0.77	***	*	C
	AT2G22870	GTP-binding protein	0.60	1.09	0.47	0.14	0.12	0.42	0.16	0.12	***		C
	AT2G27775	Expressed protein	4.60	2.47	1.26	4.52	2.56	1.97	1.91	1.73	***		C
	AT2G38750	Annexin (ANN4)	0.09	1.73	10.8	6.06	1.15	1.80	2.53	0.69	***	**	C
	AT2G38760	Annexin (ANN3)	0.12	0.91	18.6	1.52	3.5	2.16	2.53	2.20	***	***	C
	AT3G49670	LRR transmembrane protein kinase	0.85	0.88	2.50	1.75	0.17	0.47	0.34	0.42	***	***	C
	AT3G54720	Glutamate carboxypeptidase (AMP1)	0.11	0.64	1.04	1.35	0.40	0.86	1.45	1.82	***	*	C
	AT5G05860	UDP-glucuronosyl.UDP-glucosyl transferase family	0.40	0.98	1.23	1.67	0.40	1.00	1.19	2.10	***		C
	AT5G27330	Expressed protein	2.80	4.08	1.99	0.35	4.40	1.13	4.57	1.89	***	**	C
	AT5G48000	Cytochrome P450	0.35	0.88	4.11	14.57	1.26	0.27	0.86	0.86	***	***	C
	AT3g61630	ERF/AP2 member (CRF6)	1.59	0.93	0.97	3.37	0.27	0.84	0.65	1.31	***	***	D
	AT1G53885	senescence-associated protein-related	0.16	0.65	1.27	0.71	0.24	0.87	0.66	0.20	***	*	F
	AT1G80440	kelch repeat-containing F-box family	0.52	0.42	0.15	0.83	0.17	0.50	0.26	0.28	***	*	F
	AT2G24540	kelch repeat-containing F-box family	4.64	0.60	0.51	5.22	1.84	0.74	1.20	2.87	***	*	F
	AT2G35980	harpin-induced family (YLS9)	0.03	1.67	4.25	5.87	3.26	4.39	1.02	5.26	***	***	F
	AT3G01260	aldose 1-epimerase family	0.03	1.11	3.24	0.08	2.84	1.31	0.81	0.74	***	***	F
	AT3G12320	expressed protein	3.51	0.69	0.93	0.93	8.37	2.29	4.12	3.74	***		F
	AT3G27170	chloride channel (CLC-b)	2.99	1.12	1.00	0.69	2.57	1.83	1.33	2.24	***	.	F
	AT3G48520	cytochrome P450 family	0.40	0.72	0.08	0.13	0.35	0.72	0.36	0.35	***	.	F
	AT5G51720	expressed protein	0.10	0.78	0.30	1.41	0.15	0.27	0.34	0.43	***		F
	AT5G62430	Dof-type zinc finger domain-containing protein	2.67	0.28	3.58	0.58	4.55	0.96	3.07	2.17	***		F
	AT5G63450	cytochrome P450, putative	0.28	1.04	4.17	4.69	0.23	0.80	1.23	1.54	***		F
