# Supplementary material for: Transcript profiling of cytokinin action in Arabidopsis roots and shoots discovers largely similar but also organ-specific responses
Source: BMC Plant Biol. 2012 Jul 23;12:112. doi: 10.1186/1471-2229-12-112 (PMC3519560; doi:10.1186/1471-2229-12-112)
Supplement: Additional file 10 — Table S6. Cytokinin-responsive genes encoding ribosomal proteins. The table shows all cytokinin-regulated genes encoding ribosomal protein according to the GO localization database. Columns and colour-codes are the same as for Table 2. The additional column localization shows whether the protein is part of a plastid or cytosolic ribosome, according to the GO database (http://www.arabidopsis.org/tools/bulk/go/index.jsp). [file 1471-2229-12-112-S10.pdf]

Supplemental Table 5. Cytokinin-responsive genes encoding ribosomal proteins. The table shows all cytokinin-regulated genes encoding ribosomal proteins according to the localization category of the GO database (http://www.arabidopsis.org/tools/bulk/go/index.jsp). Columns and colour-codes are the same as for Table 2. The additional column "Localization" shows whether the protein is part of a plastid or cytosolic ribosome, according to the GO database. NA, information not available.

CATMA ID	AbB	Ratio Root CKX1 vs. BA0	Root BA15 vs. BA0	Root BA120 vs. BA0	Root BA1080 vs. BA0	Shoot CKX1 vs. BA0	Shoot BA15 vs. BA0	Shoot BA120 vs. BA0	Shoot BA1080 vs. BA0	FDR p-value cytokinin effect	AGI	Localization	Description
CATMA2a22440	28	1.9	1.23	0.31	2.36	0.29	0.53	0.53	0.57	1.44E-03	AT2G24090	chloroplast	ribosomal protein L35 family protein
CATMA1a27050	40	1.84	0.92	0.59	4.2	0.29	0.73	0.47	0.81	6.60E-07	AT1G29070	chloroplast	ribosomal protein L34 family protein
CATMA5a36620	34	2.42	1.74	1.06	4.23	0.32	1.14	0.53	0.75	3.06E-05	AT5G40950	chloroplast	50S ribosomal protein L27, putative (RPL27)
CATMA1a33866	25	1.28	1.87	1.02	3.32	0.37	1.07	0.73	0.82	1.60E-03	AT1G35680	chloroplast	50S ribosomal protein L21 (RPL21) (CL21)
CATMA3a14550	40	0.98	1	0.85	3.01	0.19	0.62	0.58	0.67	7.16E-06	AT3G15190	chloroplast	chloroplast 30S ribosomal protein S20
CATMA1a64680	39	0.83	0.9	0.75	1.62	0.22	0.5	0.56	0.56	2.44E-04	AT1G75350	chloroplast	ribosomal protein L31 family protein
CATMA2a41430	36	1.44	0.57	0.69	3.74	0.22	0.44	0.42	0.58	3.50E-05	AT2G43030	chloroplast	ribosomal protein L3 family protein
CATMA1a67690	35	1.6	1.29	0.98	2.73	0.29	1.01	0.85	0.7	2.94E-03	AT1G78630	chloroplast	ribosomal protein L13 family protein
CATMA1a39430	26	1.39	1.77	1.15	3.43	0.31	1.42	0.9	1.21	2.23E-04	AT1G48350	chloroplast	ribosomal protein L18 family protein
CATMA2a31610	22	1.14	0.92	0.67	2.11	0.38	0.57	0.59	0.62	1.24E-03	AT2G33450	chloroplast	50S ribosomal protein L28 (CL28)
CATMA1a53800	26	1.15	1.54	0.97	2.64	0.44	1.1	1.12	0.92	1.82E-02	AT1G64510	chloroplast	ribosomal protein S6 family protein
CATMA5a60610	17	0.89	0.61	0.39	2.73	0.17	0.38	0.39	0.48	2.65E-05	AT5G65220	chloroplast	ribosomal protein L29 family protein
CATMA3a56700	14	1.33	1.94	1.61	3.02	0.31	1.09	0.93	0.67	1.15E-03	AT3G63490	chloroplast	ribosomal protein L1 family protein
CATMA3a47150	38	1.45	0.95	0.61	2.77	0.32	0.72	0.49	0.66	1.06E-04	AT3G54210	chloroplast	ribosomal protein L17 family protein
CATMA1a06385	35	1.19	1	1.09	3.3	0.26	1.12	0.92	0.58	6.03E-03	AT1G07320	chloroplast	50S ribosomal protein L4. (CL4)
CATMA2a32000	32	0.9	1.28	0.6	3.05	0.34	0.9	0.74	0.78	1.16E-04	AT2G33800	chloroplast	ribosomal protein S5 family protein
CATMA4a36460	39	1.21	1.18	1.3	2.28	0.15	0.65	0.6	0.68	2.38E-04	AT4G34620	chloroplast	ribosomal protein S16 family protein
CATMA5a22090	40	0.3	1.21	0.57	1.36	0.16	0.74	0.4	0.43	4.28E-08	AT5G24490	chloroplast	30S ribosomal protein. putative
CATMA2a18540	6	2.5	2.26	1.59	1.84	1.45	1.91	1.25	1.51	1.27E-02	AT2G20060	chloroplast	ribosomal protein L4 family protein
CATMA2a35880	27	1.09	1.49	0.8	2.06	0.31	0.74	0.83	0.88	1.83E-02	AT2G37600	cytosol	60S ribosomal protein L36 (RPL36A)
CATMA2b38240	15	1.53	1.25	0.76	2.89	0.62	1.04	0.95	1.89	1.07E-04	AT2G40010	cytosol	60S acidic ribosomal protein P0 (RPP0A)
CATMA2a41110	21	2.55	1.14	0.64	1.88	0.63	0.54	0.78	1.32	7.76E-05	AT2G42710	cytosol	ribosomal protein L1 family protein
CATMA3a08070	35	2.91	2.98	2	3.2	0.69	1.9	1.54	1.16	3.21E-02	AT3G09200	cytosol	60S acidic ribosomal protein P0 (RPP0B)
CATMA3a53270	31	0.71	1.3	1.2	1.51	0.26	1.1	0.89	0.87	1.53E-03	AT3G60245	cytosol	60S ribosomal protein L37a (RPL37aC)
CATMA3a23320	33	0.88	2.31	1.07	1.34	0.32	1.22	1.09	1.61	2.45E-04	AT3G23390	cytosol	60S ribosomal protein L36a/L44 (RPL36aA)
CATMA3a46690	40	0.95	2.09	1.24	1.94	0.26	1.03	0.77	1.01	3.70E-06	AT3G53740	cytosol	60S ribosomal protein L36 (RPL36B)
CATMA5a21370	32	2.71	2.01	1.43	3.17	0.67	1.45	1.14	1.3	7.95E-03	AT5G23900	cytosol	60S ribosomal protein L13 (RPL13D)
CATMA4a27590	31	1.15	1.75	1.03	1.74	0.38	1.09	1.2	1.09	1.90E-02	AT4G25890	cytosol	60S acidic ribosomal protein P3 (RPP3A)
CATMA3a04590	16	1.17	3.81	2.49	1.73	0.42	2.73	2.17	1.4	1.85E-03	AT3G05560	cytosol	60S ribosomal protein L22-2 (RPL22B)
CATMA1a63670	20	1.56	2.5	1.3	2.23	0.74	1.17	1.01	1.33	8.36E-03	AT1G74270	cytosol	60S ribosomal protein L35a (RPL35aC)
CATMA1a43350	33	1.45	1.6	1.26	2.15	0.33	1.36	1.21	1.19	4.19E-03	AT1G52300	cytosol	60S ribosomal protein L37 (RPL37B)
CATMA1a55860	29	1.72	2.22	1.69	2.64	0.33	2.31	1.69	1.82	2.38E-03	AT1G66580	cytosol	60S ribosomal protein L10 (RPL10C)
CATMA1a56750	38	0.9	1.82	1.09	2.47	0.31	1.56	0.94	1.43	1.42E-05	AT1G67430	cytosol	60S ribosomal protein L17 (RPL17B)
CATMA3a49310	22	2.49	3.12	1.87	3.42	0.93	2.68	1.25	1.53	2.04E-02	AT3G56340	cytosol	40S ribosomal protein S26 (RPS26C)
CATMA3a42090	36	0.65	0.74	0.39	0.84	0.26	0.9	0.56	0.68	5.19E-03	AT3G49080	cytosol	ribosomal protein S9 family protein
CATMA5a18740	39	1.08	1.23	0.9	1.7	0.29	0.94	1.01	1.07	3.24E-04	AT5G20290	cytosol	40S ribosomal protein S8 (RPS8A)
CATMA3a10270	22	3.48	2.34	1.83	1.78	1.72	1.23	1.68	1.13	7.12E-03	AT3G11250	cytosol	60S acidic ribosomal protein P0 (RPP0C)
CATMA2a20250	37	2.53	1.53	1.07	4.23	0.77	1.49	1.27	1.63	5.63E-03	AT2G21580	cytosol	40S ribosomal protein S25 (RPS25B)
CATMA5a43680	35	4.14	2.67	1.68	3.15	1.55	3.12	3.53	2.56	5.55E-03	AT5G47700	cytosol	60S acidic ribosomal protein P1 (RPP1C)
CATMA1a00080	38	1.21	1.52	1.31	3.43	0.4	1.47	1.42	1.99	4.61E-05	AT1G01100	cytosol	60S acidic ribosomal protein P1 (RPP1A)
CATMA4a15480	38	0.77	2.15	1.09	1.39	0.26	1.36	1.62	1.37	1.25E-05	AT4G15000	cytosol	60S ribosomal protein L27 (RPL27C)
CATMA2a34850	39	0.59	1.23	0.83	0.92	0.38	1.5	1.27	1.38	1.90E-06	AT2G36620	cytosol	ribosomal protein L24 (RPL24A)
CATMA2a18270	31	1.23	2.63	1.31	1.82	0.44	2.06	1.34	1.6	1.86E-03	AT2G19730	cytosol	60S ribosomal protein L28 (RPL28A)
CATMA5a03040	33	0.99	1.44	1.18	1.43	0.3	2.46	1.73	1.17	2.86E-02	AT5G03850	cytosol	40S ribosomal protein S28 (RPS28B)
CATMA5a56410	32	1.84	2.6	1.65	1.91	1.12	2.56	3.22	1.66	5.47E-03	AT5G60670	cytosol	60S ribosomal protein L12 (RPL12C)

CATMA ID	AbB	Ratio								FDR p-value cytokinin effect	AGI	Localization	Description
		Root				Shoot							
		CKX1 vs. BA0	BA15 vs. BA0	BA120 vs. BA0	BA1080 vs. BA0	CKX1 vs. BA0	BA15 vs. BA0	BA120 vs. BA0	BA1080 vs. BA0				
CATMA4a37790	26	1.55	2.5	2.14	1.7	0.24	2.45	2.21	1.36	1.91E-03	AT4G36130	cytosol	60S ribosomal protein L8 (RPL8C)
CATMA3a01070	36	1.3	2.22	1.28	3.15	0.56	2.29	1.42	1.98	9.04E-04	AT3G02080	cytosol	40S ribosomal protein S19 (RPS19A)
CATMA4a36400	31	1.92	2.82	1.08	1.33	1.97	1.79	1.74	1.86	3.02E-02	AT4G34555	cytosol	40S ribosomal protein S25, putative
CATMA4a14640	39	1.11	1.69	0.85	1.5	0.86	2.5	1.95	2.3	2.72E-03	AT4G14320	cytosol	60S ribosomal protein L36a/L44 (RPL36aB)
CATMA5a52490	25	1.3	2.49	1.39	0.98	0.98	1.65	3.43	1.97	6.75E-03	AT5G56710	cytosol	60S ribosomal protein L31 (RPL31C)
CATMA3a13920	8	1.85	4.83	2.38	2.24	0.54	3.79	2.55	1.42	4.70E-03	AT3G14600	cytosol	60S ribosomal protein L18A (RPL18aC)
CATMA5a19900	12	4.61	4.71	2.41	1.62	2.67	2.85	3.11	2.27	3.91E-03	AT5G22440	cytosol	60S ribosomal protein L10A (RPL10aC)
CATMA5a02060	29	1.17	1.53	1.25	1.4	0.82	3.06	2.36	2.54	2.79E-02	AT5G02960	cytosol	40S ribosomal protein S23 (RPS23B)
CATMA2a18280	32	1.29	1.73	1.03	1.5	1.54	1.19	2.14	2.71	8.98E-03	AT2G19740	cytosol	60S ribosomal protein L31 (RPL31A)
CATMA2a18940	35	0.86	1.28	0.97	0.9	1	1.56	3.02	2.84	6.60E-03	AT2G20450	cytosol	60S ribosomal protein L14 (RPL14A)
CATMA1a23910	27	0.8	1.9	1.2	1.26	1.69	1.92	2.65	2.4	1.15E-03	AT1G25260	cytosol	acidic ribosomal protein P0-related
CATMA3a45970	17	0.93	2.06	1.1	1.14	1.88	3.6	7.11	6.01	3.43E-03	AT3G53020	cytosol	ribosomal protein L24 (RPL24)
CATMA1a54180	11	0.63	2.58	1.93	0.34	2.76	1.4	5.12	0.95	4.61E-06	AT1G64880	cytosol	ribosomal protein S5 family protein
CATMA1a14860	38	1.25	0.64	0.5	2.22	0.28	0.37	0.47	0.77	6.89E-04	AT1G15810	NA	ribosomal protein S15 family protein
CATMA5a50910	37	0.99	0.74	0.97	3.04	0.38	0.94	0.54	0.98	8.11E-05	AT5G55140	NA	ribosomal protein L30 family protein
CATMA5a35430	22	1.82	2.75	3.15	1.06	1.28	0.96	1.89	0.68	3.01E-04	AT5G39785	NA	expressed protein
CATMA5a25250	15	3.24	2.8	1.18	0.97	3.71	0.87	1.66	1.41	3.83E-03	AT5G27820	NA	ribosomal protein L18 family protein
CATMA5a21050	27	2.72	2.02	1.09	1.73	1.92	2.37	1.69	1.73	2.81E-02	AT5G23535	NA	KOW domain-containing protein
CATMA4a08100	7	0.2	0.83	1.55	0.33	2.64	0.77	3.29	1.05	1.69E-03	AT4G08350	NA	KOW domain-containing transcription factor family protein
