# Supplementary material for: Transcript profiling of cytokinin action in Arabidopsis roots and shoots discovers largely similar but also organ-specific responses
Source: BMC Plant Biol. 2012 Jul 23;12:112. doi: 10.1186/1471-2229-12-112 (PMC3519560; doi:10.1186/1471-2229-12-112)
Supplement: Additional file 11 — Table S7. Primer sequences used for qRT-PCR. The primer sequences were selected using GENOPLANTE™S.P.A.D.S. (http://www.psb.ugent.be/SPADS/, grey background) or NCBI Primer-BLAST (http://www.ncbi.nlm.nih.gov/tools/primer-blast/). [file 1471-2229-12-112-S11.pdf]

Supplemental Table 7. Primer sequences used for qRT-PCR. The primer sequences were selected using GENOPLANTE™S.P.A.D.S. (http://www.psb.ugent.be/SPADS/, grey background) or NCBI Primer-BLAST (http://www.ncbi.nlm.nih.gov/tools/primer-blast/).

AGI	Gene name	Forward primer	Reverse primer
AT1G19050	ARR7	CTTGGAACCAATCTGCTCTC	ATCATCGACGGCAAGAAC
AT1G28370	ATERF11	CCGCTCGTGCCTACGACAAA	TGCTCTGGCTCAGGCTCTGT
AT1G67110	CYP735A2	TGCTAACGAAGCACAATCCGGTC	GAAAGCCGGAGCTGCCATGT
AT1G75820	CLV1	CCATGATTGGTCCTAAAGGACACGG	AACACGAGCATCGTCGTCACA
AT1G77640	ERF013	CGACGCTGCTCTCTTGTGTCTT	TCGGACGGAGGGGTAAAATGGT
AT1G79040	PsbR	AGAGGATTACCGTCGCTCACAAGA	ACACCGTATCCCTTGCCCTTTCT
AT2G34490	CYP710A2	GCATCGTAACGTATCCGAACCTCTG	TCACCTCACGAGACACATCACGA
AT2G34500	CYP710A1	AGGCGCGTCGCAAAGTATCC	AACACTCGTGGGCCACTCAC
AT2G40670	ARR16	TCAGGAGGTTCTTGTTCGTCTT	AACCCAAATACTCCAATGC
AT2G47460	MYB12	TGCCTTGACTTTTGTGGTCAGTGG	ACCGCCCTCTCTTGATACCGA
AT3G15990	SULTR3;4	TGGGTCATGGCACTAACA	CTGTTTTCTTCGGCGGTA
AT3G26570	PHT2;1	GCTGGTGCCGTTTTCTGGAGTT	CCGCTGCTTGTCCTGGGTTT
AT4G08300	T12G13.140	GCACACTTGCGGCTGTTTACTC	GCCAAAACTAACACGCCGAGGA
AT4G10310	HKT1	ATCAGCGATGGTGGCTGCAA	GCGGCCAGATTTGGCTGTGA
AT4G29740	CKX4	GCTCCAGGGTTTATGTTCGT	GCGGTTGTTCCATTTGTTTC
AT5G07580	MBK20.1	CCTACCCCATCCATGGCTGCTA	CAGAGCTTTCCTCAAAACTCGCCA
AT5G47450	TIP2;3	TTGGCTCCATCGTCGCTTGTC	TGACGACACCTTCGACCGCT
AT5G51190	ERF105	GCCAACGCAAACCACCTCTTG	GCCTAACCAGACACGAACACCTT
AT5G57090	PIN2	AGGAAACAGCAGATGCCGCC	CAAGGGACCAAGCAAGGCCA
AT5G65990	K2A18.5	AGATCCTGACATCCAAGGC	TCCATGGTGCGTCTTTCT
